# Nasopharyngeal microbiota composition of children is related to the frequency of upper respiratory infection and acute sinusitis

**DOI:** 10.1186/s40168-016-0179-9

**Published:** 2016-07-01

**Authors:** Clark A. Santee, Nabeetha A. Nagalingam, Ali A. Faruqi, Gregory P. DeMuri, James E. Gern, Ellen R. Wald, Susan V. Lynch

**Affiliations:** Division of Gastroenterology, Department of Medicine, University of California San Francisco, San Francisco, CA 94143 USA; Department of Pediatrics, University of Wisconsin School of Medicine and Public Health, Madison, WI 53792 USA; Present address: Janssen Prevention Center, 2 Royal College Street, London, NW1 0TU UK

**Keywords:** Microbiota, Upper respiratory infection, URI, Acute sinusitis, Pediatrics, Children

## Abstract

**Background:**

Upper respiratory infections (URI) and their complications are a major healthcare burden for pediatric populations. Although the microbiology of the nasopharynx is an important determinant of the complications of URI, little is known of the nasopharyngeal (NP) microbiota of children, the factors that affect its composition, and its precise relationship with URI.

**Results:**

Healthy children (*n* = 47) aged 49–84 months from a prospective cohort study based in Wisconsin, USA, were examined. Demographic and clinical data and NP swab samples were obtained from participants upon entry to the study. All NP samples were profiled for bacterial microbiota using a phylogenetic microarray, and these data were related to demographic characteristics and upper respiratory health outcomes. The composition of the NP bacterial community of children was significantly related prior to the history of acute sinusitis (*R*^2^ = 0.070, *P* < 0.009). History of acute sinusitis was associated with significant depletion in relative abundance of taxa including *Faecalibacterium prausnitzii* and *Akkermansia* spp. and enrichment of *Moraxella nonliquefaciens*. Enrichment of *M. nonliquefaciens* was also a characteristic of baseline NP samples of children who subsequently developed acute sinusitis over the 1-year study period. Time to develop URI was significantly positively correlated with NP diversity, and children who experienced more frequent URIs exhibited significantly diminished NP microbiota diversity (*P* ≤ 0.05).

**Conclusions:**

These preliminary data suggest that previous history of acute sinusitis influences the composition of the NP microbiota, characterized by a depletion in relative abundance of specific taxa. Diminished diversity was associated with more frequent URIs.

**Electronic supplementary material:**

The online version of this article (doi:10.1186/s40168-016-0179-9) contains supplementary material, which is available to authorized users.

## Background

Upper respiratory infection (URI) is one of the most common reasons for children in the USA to seek medical care [1]. URIs frequently lead to bacterial complications, resulting in billions of dollars in health care expenditures [2]; 30 % of URIs are complicated by acute otitis media and 8 % by rhinosinusitis [2]. The presence of *Streptococcus pneumoniae*, *Haemophilus influenzae*, and/or *Moraxalla catarrhalis* in nasopharyngeal (NP) cultures from children with URIs is predictive of increased likelihood of progression to acute otitis media [2], and these species are also known to potentiate the effect of rhinovirus infection on the likelihood of asthma exacerbation [3].

A cross-sectional study of children and adults (*n* = 51 children, aged 1–4.5 years; *n* = 19 adults) in Canada demonstrated that, like the gastrointestinal microbiota [4], changes in NP bacterial microbiota occur with increasing age; less diverse communities are observed in younger children and more even, diverse communities are characteristic of adults [5]. The beta-diversity of bacterial communities was more similar in adults compared to that observed in children, indicating greater heterogeneity in upper airway microbiota in early life [5]. Teo et al. also recently demonstrated, using longitudinally obtained NP samples collected over the first year of life from a cohort of 234 Australian infants, that the NP microbiota undergoes compositional development during this period [6]. Subjects were grouped into one of six bacterial community states, each dominated by a distinct genus (*Moraxella*, *Haemophilus*, *Streptococcus*, *Corynebacteria*, *Alloiococcus*, or *Staphylococcus*) [6]. Infants whose NP microbiota were dominated by *Moraxella*, *Haemophilus*, or *Streptococcus* at the time of viral respiratory infection exhibited significantly higher risk for developing lower airway infection. In addition, early colonization with a *Streptococcus*-dominated community conferred an increased risk for the development of persistent asthma [6].

A separate study of the NP bacterial microbiota of 200 Dutch children sampled at 1.5, 6, 12, and 24 months identified eight distinct bacterial community profiles in this population [7]. While some of these community states exhibited compositional evolution over time, others remained stable over the 2-year study period. For example, *Haemophilus*-dominated communities never persisted for more than one time-point and exhibited a high magnitude of temporal compositional instability, while *Moraxella* or *Corynebacterium*/*Dolosigram* dominated communities were typically stable and also were associated with fewer parental reported URIs [7]. These observations indicate that the composition of the pediatric upper airway represents a critical factor that may either potentiate or protect against infection by respiratory pathogens. They also indicate that the interplay between the bacterial microbiota and respiratory pathogens associated with upper airway infection is important to consider. Both bacteria and viruses can influence each other’s pathogenicity [8] and a number of interactions between specific viruses and bacterial species have been reported in the airways [9, 10]. For example, human rhinovirus infection was found to significantly increase the binding of *Staphylococcus aureus*, *S. pneumoniae*, or *H. influenzae* to primary human nasal epithelial cells [11] and in an independent study of human bronchial epithelial cells, pre-incubation with *S. pneumoniae* significantly increased infection by human metapneumovirus while pre-incubation with *H. influenzae*, *S. aureus*, or *M. catarrhalis* had no effect [12].

The nuances of these relationships, especially in the context of the complex microbial communities of the upper airway (in both uncomplicated URI as well as acute sinusitis), are not completely understood. In this study, we sought to identify variables that influence the composition of the NP microbiota of healthy children and determine whether features of these communities are related to susceptibility to URI or acute sinusitis in a North American cohort of 49–84-month-old subjects.

## Results

### Variability in NP microbiota composition is significantly associated with a history of acute sinusitis

**Fig. 1:**
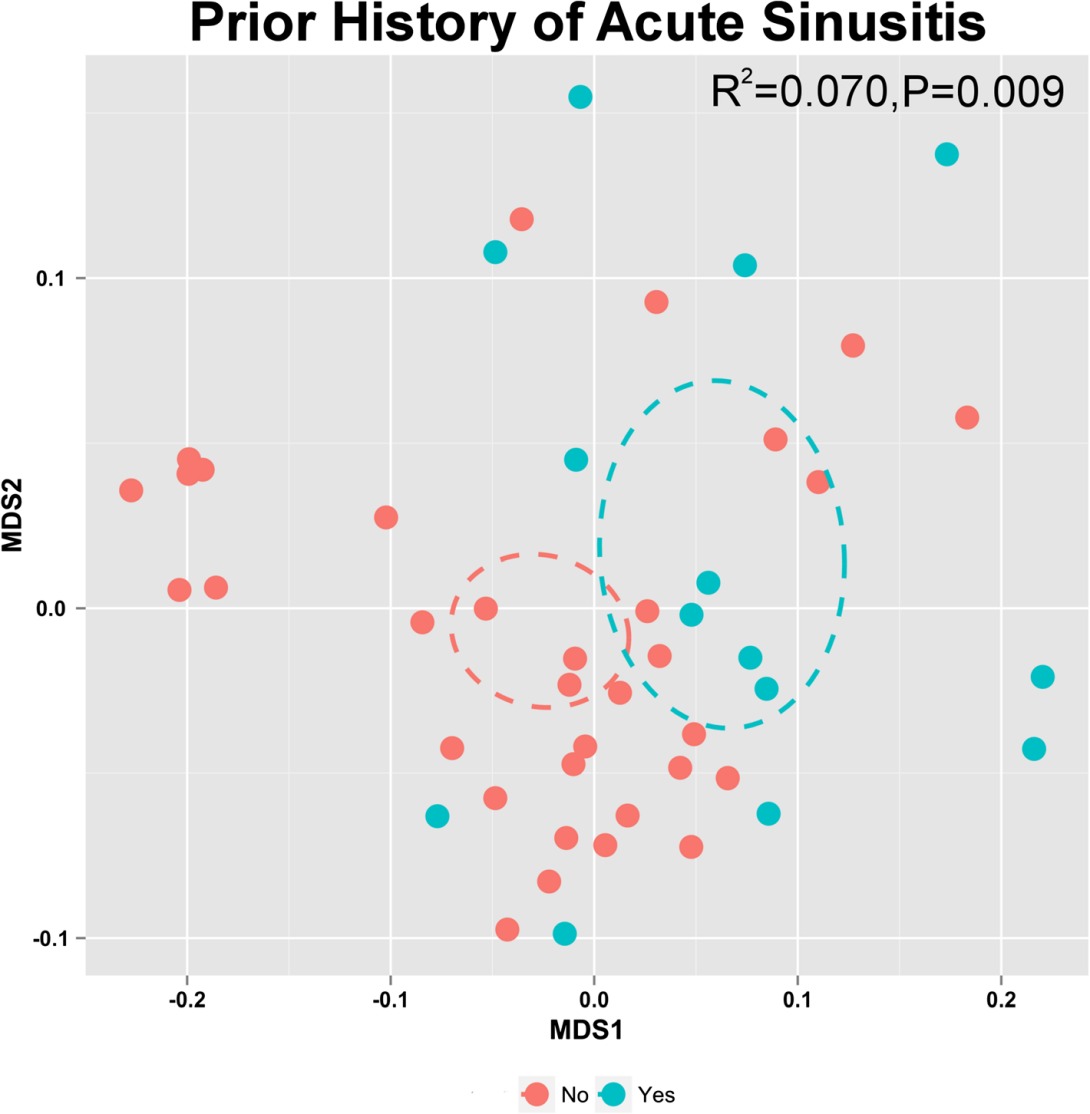
Permutation testing of non-metric dimensional scaling based on a Canberra distance matrix identifies acute sinusitis history (yes *n* = 14, no *n* = 33) as significantly (*P* ≤ 0.01) related to the observed variation in NP bacterial community composition upon entry to the study. Ellipses indicate 95 % confidence interval, stress = 0.1226

A total of 951 taxa were identified in baseline NP microbiota of participants (*n* = 47) in our cohort. These bacterial communities were variably composed of members of the *Rickenellaceae*, *Lachnospiraceae*, *Verrucomicrobiaceae*, *Pseudomonadaceae*, and *Moraxellaceae* as well as multiple unclassified members of the phylum *Proteobacteria*. To identify demographic features that might explain the observed variation in nasopharyngeal bacterial community composition (for demographic information see Additional file 1: Table S1), a pair-wise Canberra distance matrix was constructed and used in permutational multi-variate regression analyses against a total of 58 study variables (Additional file 1: Table S2). History of acute sinusitis (chronic sinusitis was an exclusion criterion) prior to entry into the study exhibited a strong significant relationship (Adonis, *R*^2^ = 0.070; *P* < 0.009) with bacterial community membership in our cohort (Fig. 1, Additional file 1: Table S2).

Our study used independent NP samples collected from individual participants over a 12-month study period that spanned all four seasons (see Additional file 2: Figure S1 for sampling schematic). Season of sample collection also demonstrated a relationship with bacterial beta-diversity (Adonis, *R*^2^ = 0.137, *P* < 0.006). However, samples were analyzed in batches as they were collected, and we noted that a significant relationship between batch and season existed (Fisher’s exact test *P* = 8.3 × 10^−9^) which confounded this observation. A similar analysis did not find a significant relationship between batch and on the history of acute sinusitis (Fisher’s exact test *P* > 0.12).

**Fig. 2:**
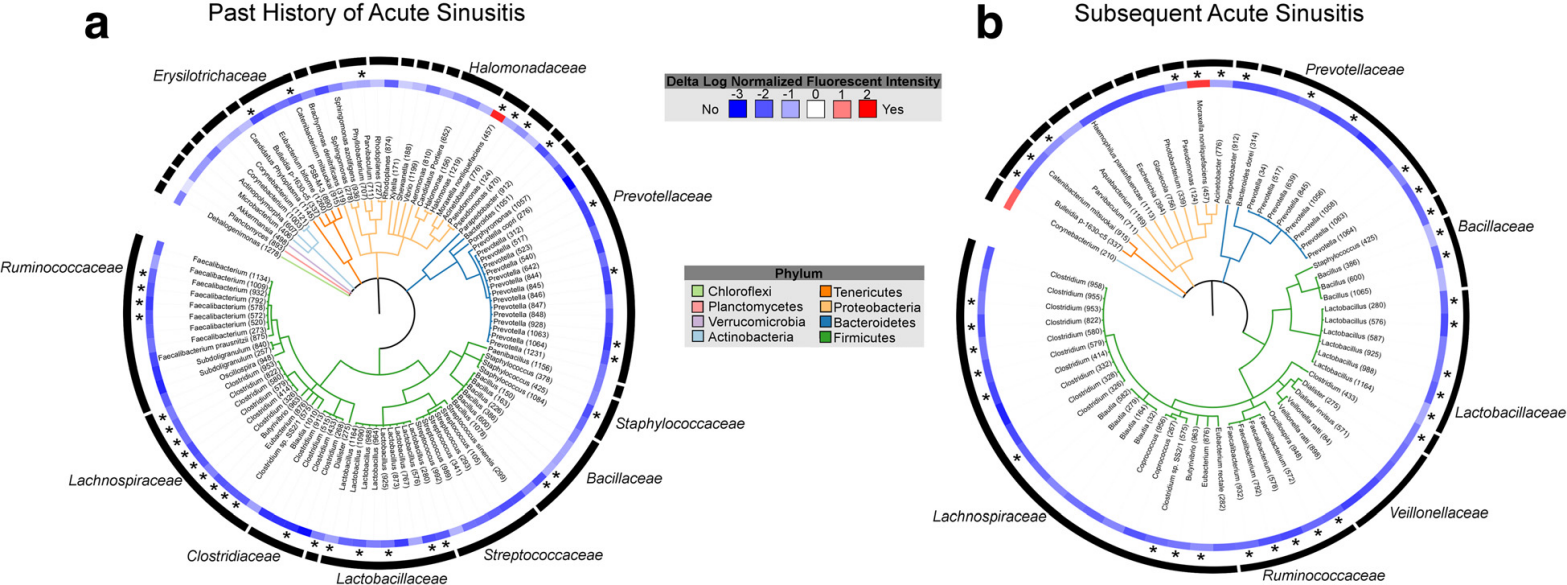
Phylogenetic trees displaying genera that exhibit significant differences in relative abundance across scomparison groups. **a** Children with a history of acute sinusitis (*n* = 14) have NP microbial communities significantly (Welch’s *t* test; *P* < 0.05, *q* < 0.05) enriched (*red*) for *Moraxella nonliquefaciens* and depleted (*blue*) of 100 genera compared with those without a history of sinusitis (*n* = 33). **b** Children who developed acute sinusitis during the 1-year period following baseline sampling (*n* = 7) have communities that are significantly (Welch’s *t* test; *P* ≤ 0.05, *q* < 0.25) enriched for *Moraxella nonliquifaciens* and *Corynebacterium* spp. (*red*) and depleted of 59 genera (*blue*) relative to children who did not develop acute sinusitis (*n* = 33). Branches are colored by phylum. Numbers specifying unique eOTUs within each genus are shown in *parentheses*; eOTUs consistently identified across both comparisons are indicated with an *asterisk*

Compared with children who had no history of acute sinusitis (*n* = 33), those with a past history of acute sinusitis (*n* = 14) did not exhibit differences in α-diversity indices (Wilcoxon rank-sum test *P* > 0.5 for richness, Inverse Simpson, or Faith’s phylogenetic diversity), suggesting that differences in microbiota characterizing these groups may be due to the enrichment or depletion of a subset of taxa within these bacterial communities. A total of 309 taxa (representing 101 genera) exhibited significant differences in relative abundance between children with and without a history of acute sinusitis. NP samples from children with a prior history of acute sinusitis were characterized by significant depletion of 308 of the 309 taxa, including those represented by *Akkermansia*, *Faecalibacterium prausnitzii*, *Clostridium*, *Lactobacillus*, *Prevotella*, and *Streptococcus* species. The only taxon that exhibited a significant (Welch’s *t* test, *P* < 0.05, *q* < 0.05) increase in relative abundance in these subjects was represented by *Moraxella nonliquefaciens* (Fig. 2a; Additional file 1: Table S3; Additional file 3: Figure S2A).

Based on the relationship between a prior history of acute sinusitis and composition of the microbiota, we next examined differences in the relative abundance of baseline taxa associated with the development of acute sinusitis subsequent to baseline sampling (occurring during the 1 year clinical follow-up period; *n* = 33 healthy, *n* = 7 subsequent sinusitis). Subsequent acute sinusitis was associated with substantial taxon depletion (*n* = 143) and again included those belonging to *Faecalibacterium*, *Clostridium*, *Lactobacillus*, and *Prevotella*. Children who subsequently developed acute sinusitis displayed significant enrichment (Welch’s *t* test, *P* ≤ 0.05, *q* < 0.25) of *Moraxella nonliquefaciens* (Fig. 2b; Additional file 1: Table S4; Additional file 3: Figure S2B) and *Corynebacterium* spp. (Fig. 2b; Additional file 1: Table S4) in their baseline NP microbiota. It should be noted that this is a relatively high *q* value cut-off (*q* < 0.25) due to the small number of children (*n* = 7) who developed sinusitis during our study period, hence larger cohorts are needed to validate this finding. A large majority of the empiric Operational Taxonomic Units (eOTUs) depleted in subjects with a history of acute sinusitis were also depleted in children who developed acute sinusitis subsequent to baseline sampling, including multiple members of the *Lactobacillus* genus (Fig. 2b; Additional file 1: Table S4). Of note, a prior history of acute sinusitis was not significantly associated with the development of a subsequent episode of acute sinusitis following baseline sampling (Fisher’s exact test, *P* = 0.34), indicating that distinct groups of subjects were included in these analyses and that the taxonomic depletions and enrichments associated with acute sinusitis are consistent features of the disease.

### Compositional differences based on URI frequency

**Fig. 3:**
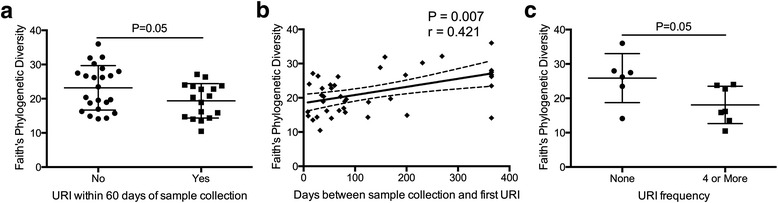
**a** Children who experience at least one URI within 60 days of sample collection (*n* = 17) exhibit significantly lower phylogenetic diversity than those who do not (*n* = 23) experience any URI within this time period (Welch’s *t* test, *P* = 0.05; Shapiro-Wilk test *P* > 0.17). **b** Increased phylogenetic diversity is significantly correlated with a greater number of days between sample collection and first subsequent URI (Spearman Correlation, *r* = 0.421, *P* = 0.007). Trendline and confidence intervals indicate that this relationship is also significant based on regression analysis (*F* test; *R* ^2^ = 0.233; *P* = 0.0016). **c** Children with four or more URIs (*n* = 13) exhibit significantly lower phylogenetic diversity than those who experienced no URIs (*n* = 8) over the 12-month clinical monitoring period (Wilcoxon rank-sum test, *P* = 0.05)

We next examined whether features of the baseline composition of the NP microbiota were associated with susceptibility to URI by examining samples from 40 subjects (7 of 47 subjects were lost to follow-up) who reported a range of URI frequency (from 0–9; mean of 2.25) within the 12-month clinical follow-up  period. Bacterial community richness, Shannon diversity, Inverse Simpson’s diversity, and Faith’s phylogenetic diversity indices were all significantly negatively associated with the frequency of URIs (negative binomial regression; *P* < 0.05; Table 1). Children who experienced at least one URI (*n* = 17) within 60 days of collection of the baseline sample had significantly lower phylogenetic diversity (Fig. 3a; Welch’s *t* test, *P* = 0.05; Shapiro-Wilk test *P* > 0.17) compared to those who had no URIs within that time frame (*n* = 23). Time to development of URI, defined as the number of days between the collection of the baseline sample and the first incidence of URI (a value of 365 days was assigned to those children who did not experience a URI during the year of monitoring), was also significantly correlated with phylogenetic diversity (Fig. 3b; Spearman Correlation, *r* = 0.421, *P* = 0.007). In addition, significantly decreased phylogenetic diversity (Fig. 3c; Wilcoxon rank-sum test, *P* = 0.05) was also observed among children who experienced a high frequency of URI (four or more URI, *n* = 13) relative to children who did not experience any URI (*n* = 8) over the 1-year study period following sample collection. Hence, these data indicate that diminished diversity of the NP microbiota is a precursor to URI in these children.

**Table 1:** Negative binomial modeling indicates that NP bacterial alpha-diversity indices are inversely related to frequency of URI (*n* = 40)

Diversity index	Estimate	*P*	Goodness of fit (*X* ^2^)
Shannon diversity index	−0.475	0.040	0.27
Richness	−0.004	0.045	0.28
Inverse Simpson’s index	−0.004	0.044	0.28
Faith’s phylogenetic diversity index	−0.050	0.023	0.29

## Discussion

Previous studies in a cohort of 234 Australian infants (up to 12 months of age) have demonstrated relationships between very early-life NP microbiota, characteristically dominated by specific bacterial genera, and respiratory illness [6]. Here we report similar relationships between acute sinusitis, URI, and the NP microbiota of children between 49 and 84 months of age in a US cohort using a distinct platform to generate bacterial microbiota profiles. Specifically, we identify a taxon represented by *M. nonliquefaciens* as significantly enriched both in children who report a past history of acute sinusitis and among those who developed sinusitis following baseline sampling, implicating this organism as potentially important in the pathogenesis of sinus infection. Teo and colleagues have previously demonstrated that *Moraxella* can stably colonize the microbiota of the infant NP, with 31 % of their cohort exhibiting this genus as the dominant member of the NP bacterial community [6]. In addition, the majority of infants colonized with *Moraxella* experienced a subsequent acute respiratory infection prior to the collection of additional samples within that study (samples were obtained at 2, 6, and 12 months of age) [6]. The authors also found that NP communities dominated by *Moraxella* (as well as those dominated by *Streptococcus or Haemophilus*) are also associated with significantly more frequent acute respiratory infections compared to healthy NP samples, even after adjusting for a large set of potential confounders (age, gender, season, number of prior infections, antibiotic intake, mother’s antibiotic intake, delivery mode, and breastfeeding). An independent study of children aged 4 to 12 years (*n* = 308) also demonstrated that those with upper airway microbiota significantly enriched for *Moraxella catarrhalis* experienced more severe and protracted symptoms of viral URI [3]. In contrast, Biesbroek and colleagues found that children with *Moraxella*-dominated NP bacterial communities suffered fewer URIs in 6 months following sample collection [7]. These apparently conflicting data underscore the limitations of biomarker gene sequencing approaches, which frequently fail to discriminate between discrete species or strains of a given genus, and provide no information on function of these organisms in the context of the specific microbial species with which they co-associate. In addition, previous studies have also demonstrated substantial variation in virulence capacity across members of the same species [13, 14] including *Moraxella catarrhalis* [15, 16], suggesting that distinct *Moraxella* species or strains may elicit dichotomous outcomes in pediatric patients. Hence, metagenomic studies of the functional genetic capacity as well as comparative genomic studies of *Moraxella* isolates are necessary to identify factors that potentiate the effect of upper airway infection and enhance or prolong respiratory symptoms. Such efforts may lead to improved prediction of susceptibility to infection.

Our data did not find a relationship between URI frequency and *Moraxella* enrichment; what was apparent, however, was an association between this genus and acute sinusitis. Since the study by Biesbroek and colleagues used retrospective parent-reported mild respiratory tract infections as outcomes, the role of *Moraxella* in a more severe infection or sinusitis outcomes cannot be assessed in that study [7].

In addition to *Moraxella*, a *Corynebacterium* was enriched in relative abundance in the NP microbiota of children who experienced acute sinusitis subsequent to baseline sample collection during the study period. The small numbers in this study and borderline false discovery correction values mandate caution in the interpretation of these findings. However, Abreu et al. previously found *Corynebacterium tuberculostearicum* to be significantly enriched in the maxillary sinuses of adults with chronic rhinosinusitis compared to healthy control subjects [17]. The authors subsequently confirmed the ability of *C. tuberculostearicum* to induce acute sinusitis in the context of an antimicrobial-depleted murine model of sinus infection. Moreover co-installation of *Lactobacillus sakei* (one of a number of taxa acutely depleted in relative abundance among chronic rhinosinusitis patients) protected animals against *C. tuberculostearicum* infection [17]. Our pediatric data exhibits similarity with these murine studies, in that six members of the *Lactobacillus* genus were among those taxa most significantly depleted in relative abundance in the NP bacterial communities of children who developed sinusitis during our study. Five of these same taxa were also depleted in relative abundance in the NP microbial communities of children with a prior history of sinusitis. In addition to *Lactobacillus*, many other bacterial taxa including *Akkermansia*, *Faecalibacterium prausnitzii*, *Clostridium*, *Prevotella*, and *Streptococcus* species were depleted in relative abundance among children with a prior history of acute sinusitis. Though traditionally associated with gut microbiota, anaerobic bacterial species can exist in biofilms in the upper respiratory tract [18] and *Akkermansia* and *Faecalibacterium* have previously been detected in the nasopharynx of children [19, 20]. While its role in the airway is unknown, gastrointestinal *Akkermansia muciniphilia* metabolizes mucin and has been shown to activate immune homeostasis, increasing host expression of antimicrobial peptides such as RegIII_γ_ and improving barrier function via an increase in 2-oleoylgylcercerol [21–23]. However, whether such mechanisms play a role at the airway mucosal surface remains to be determined. Mechanisms by which *Lactobacillus* and other bacterial species depleted in the NP microbiota of sinusitis patients may prevent the development of disease include competitive exclusion of pathogenic species. A previous murine study indicated that intra-nasal inoculation of mice with *L. fermentum* decreased *S. pneumoniae* burden throughout the respiratory tract and increased the number of activated macrophages in the lung and lymphocytes in the tracheal lamina propria [24]. Hence, it is plausible that the absence of NP genera with known competitive exclusion and immunomodulatory capabilities leads to pathogen expansion and associated clinical manifestations of upper airway infection.

Permutational analyses indicated that the composition of the NP microbiota is significantly associated with the season of sample collection. While this observation in our study was confounded by a batch effect, our finding corroborates those of an Australian infant cohort, indicating that the composition of the NP microbiota in the first year of life is related to season [6]. Seasonality has previously been related to respiratory illness [25], and in our study, we demonstrate that increased NP diversity is associated with protection against URI. These data raise the intriguing possibility that the composition of the microbiota of the upper airway is related both to local climactic conditions and seasonal susceptibility to upper airway infection. However, studies involving repeated measures from individual participants are necessary to corroborate our observations and to determine composition dynamics of the NP microbiota that contribute to this phenomenon.

Limitations of our study include limited ability to discern causal connections between enriched Operational Taxonomic Units (OTUs) and sinusitis history. We do show that a history of sinusitis, its pathophysiology or treatment, may shape the NP microbiota—which may inform future studies and their design. Additionally, though we recognize that the composition of the microbiota in the upper airways is likely highly influenced by antibiotic administration, obtaining a complete history of previous antibiotic administration is difficult and often imprecise due to poor adherence and transition across multiple medical providers and systems and thus was not examined in this study. The pervasive effects of antimicrobials on the human microbiota are well-described [26, 27], and it is likely that lifetime antibiotic use plays an important role in shaping the baseline NP microbial community. However, such studies are best suited to birth cohorts, where this information is collected prospectively and can be related retrospectively both to microbiota composition and health outcomes. This study used the 16S rRNA PhyloChip, rather than the next generation 16S rRNA gene sequencing. The microarray platform offers the advantage of detecting OTUs in parallel, allowing for the detection of rare, low abundance organisms that may not be detected by shallower sequencing efforts [28]. However, the PhyloChip is limited in only determining differences in relative abundance within each OTU and cannot provide insight into community structure, absolute abundance of OTUs, or discover novel OTUs. Several studies have, however, validated the application of this assay, demonstrating consistency in findings generated by this approach and parallel sequencing of the same samples [28, 29]

## Conclusions

The composition of the NP microbiota in healthy children between 49 and 84 months of age is associated with past and subsequent history of acute sinusitis and frequency of URI. Widespread bacterial taxon depletion and enrichment of *M. liquefaciens* and *C. tuberculostearicum* are associated with upper airway infection and the development of acute sinusitis. Collectively, these findings provide evidence of close connections between microbial colonization of the airways and susceptibility to upper respiratory illnesses in early childhood and raise the possibility that the manipulation of the airway microbiota could be applied to the prevention of childhood respiratory illnesses.

## Methods

### Study population

Forty-seven healthy children aged 49–84 months were recruited during well-child visits from two primary care clinics in Madison, WI, for a prospective, longitudinal cohort study. This study was approved by the University of Wisconsin Institutional Review Board. Informed parental (or legal guardian) written consent was obtained as well as assent from older children (signed consent documentation was retained as part of the patient’s file). Information on demographic variables, pets in the household, medical history, and various exposures (e.g., farm animals, medications; Additional file 1: Table S1) was collected. Children were enrolled between February 2012 and February 2013 and followed for 1 year (Additional file 2: Figure S1). Seven children discontinued participation before completing a full year of the study due to discomfort with the sampling procedure. NP surveillance samples were obtained from the children at entry to the study (*n* = 47); enrollment samples were collected from participants across all four seasons (defined by equinox and solstice dates: spring commencing on March 20th, summer, June 20th, fall, September 22nd and winter, December 21st) [30]. Longitudinal clinical follow-up was conducted for 12 months following entry into the study and baseline sample collection. To monitor URI frequency, parents were instructed to call the study nurse when respiratory symptoms persisted for at least 48 h and record their children’s respiratory symptoms. Parents were also contacted monthly by the study team to determine the occurrence of episodes of infection, which they may have previously failed to report. URI was defined as nasal congestion, nasal discharge, or cough (with or without fever) lasting 48 hours or more. Sinusitis was defined in one of three ways: (1) respiratory symptoms (including nasal discharge or cough or both) having lasted more than 10 days and not improving, (2) a combination of purulent (thick, colored, and opaque) nasal discharge plus temperature >39 °C for at least 72 h, or (3) sudden worsening of a viral URI after apparent improvement usually beyond the sixth day of illness [31]. The medical record was investigated to determine if there were unreported instances of antimicrobial use. Children were excluded from the study if they had a congenital or acquired immunodeficiency, craniofacial abnormalities, cystic fibrosis, allergic rhinitis, received antibiotics in the past 30 days, developed a URI within 1 week of baseline sampling, or a previous diagnosis of chronic sinusitis.

### Sample processing

A flocked swab was placed into the nasopharynx and rotated. The swab was cut off with sterile scissors and placed into sterile DNAase/RNase-free cryovials containing 2 ml of RNALater (Ambion). Samples were collected at the University of Wisconsin, Madison, and stored at 4 °C for 24 h to permit preservative to penetrate cells, prior to freezing at −80 °C and then shipped in batches to the University of California San Francisco for microbiota analysis. DNA was extracted using a combination of bead beating and the AllPrep kit (Qiagen) as previously described [17]. Extracted DNA was used for high-resolution microbiota profiling using the 16S rRNA PhyloChip (Second Genome) and TaqMan qPCR. To assess relationships between environmental exposures or URI and NP microbiota, a single baseline sample from each child (*n* = 47) collected during a healthy (non-URI) period was used.

### Microbiota profiling

Microbiota profiling with 16S rRNA PhyloChip (version G3) which can detect up to 60,000 bacterial and archeal taxa was performed as previously described [32, 33]. Briefly, extracted DNA was used as template (30 ng) in eight PCR reactions which run across a gradient of annealing temperatures (48.7–57.1 °C). Amplicons were pooled per sample, gel purified, and quantified with Alpha Ease software version 4.1.0 (Alpha Innotech Corporation) using E-gels (Invitrogen). A total of 250 ng of fragmented, biotin-labeled 16S rRNA product per sample was applied to each PhyloChip. Detection and quantification criteria for each OTU detected by PhyloChip were defined using the empiric Operational Taxonomic Unit approach as previously described [34]. Briefly, groups of probes are assigned to a specific eOTU based on taxonomic relatedness as well as their correlation in fluorescent intensity throughout a given experiment and then taxonomically annotated by a Bayesian method based on the sequence of the probes within each eOTU probe grouping. An 80 % certainty cut-off was used for taxonomic annotation. A negative control sample was carried through from extraction to microbiota profiling on the PhyloChip; a total of seven eOTUs were detected in this sample (see Additional file 1: Table S5). These eOTUs were removed from the dataset and not considered in data analyses.

### Viral detection

Viral detection by the PCR-based Respiratory Multicode Assay (EraGen Biosciences) [35] as well as human rhinovirus detection by PCR [36] was performed as previously described.

### Bacterial burden

Total 16S copy number was determined as previously described using TaqMan universal PCR Master mix (Applied Biosystems) with the following primers: P981F, 5′-TGGAGCATGTGGTTTAATTCGA; P1033R, 5′-TGCGGGACTTAACCCAACA; UniProbe, 5′-CACGAGCTGACGACARCCATGCA [37]. To determine if bacterial burden had an effect on the observed variation in the bacterial community composition of the nasopharynx, a pair-wise Canberra distance matrix was constructed and used in permutational multi-variate regression analyses and no significant relationship was found (Adonis, *P* = 0.529, *R*^2^ = 0.050).

### Statistical analysis

Much of the statistical analyses were performed using custom scripts and packages in the R statistical environment as we have previously described [33]. Faith’s phylogenetic diversity is defined as “the sum of the lengths of all those branches that are members of the corresponding minimum spanning path”; the tree referring to a bacterial phylogenetic tree was calculated using QIIME [38, 39]. All other diversity indices were calculated using the *Vegan* (http://vegan.r-forge.r-project.org/) package in the R statistical environment. The indices used are defined as follows: Richness is the total number of unique OTUs; Inverse Simpson’s index is 1/*D* = ∑ (*n*/*N*)^2^, where *D* = Simpson’s index, *n* = the total number of organisms of a particular OTU, and *N* = the total number of organisms of all OTUs; Shannon’s diversity is calculated by the equation *H* = −∑*(*p*_*i*_ ln(*p*_*i*_)), where *H* = Shannon’s index and *p* is the proportional abundance of OTU *i*.

Fluorescence intensities were used to calculate weighted, pair-wise Canberra distances with the following equation: *D*_*jk*_ = (1/NZ) * Σ^All Non-Zero OTUs^(|*x*_*ij*_ − *x*_*ik*_|/(*x*_*ij*_ + *x*_*ik*_)) where *j* = sample 1, *k* = sample 2, NZ = the number of OTUs with fluorescence intensities greater than 0 in at least one sample, *x* = fluorescence intensity, and *i* = OTU. Canberra distance calculates pair-wise distances, based on the average absolute difference in abundance between OTUs present normalized to the combined OTU abundance. Canberra distances were visualized using non-metric multi-dimensional scaling to illustrate community dissimilarity. Negative binomial regression was used to assess trends in microbiota alpha-diversity relative to URI frequency [40]. As previously described, two tailed Welch’s *t* tests adjusted for false discovery with *q* values were used on log-normalized fluorescence data to assess differences in taxon relative abundance between specific groups (an underlying normal distribution of probe set fluorescent intensities is assumed) [17, 33, 41]. Because each probe set (corresponding to a given eOTU) is composed of a group of oligonucleotides with different sequences and therefore different hybridization affinity with their target 16S rRNA, comparisons in relative abundance cannot be made across eOTUs, within a bacterial community. Prism version 6.0 (GraphPad Software, www.graphpad.com) was used to construct figures and perform statistical tests. The Interactive Tree of Life (iTOL, http://itol.embl.de) was used to construct and visualize bacterial phylogenetic trees [42].

## Abbreviations

eOTU, empiric operational taxonomic unit; NP, nasopharyngeal; OTU, operational taxonomic unit; URI, upper respiratory infection

## Additional files

Additional file 1: Table S1. Summary table of demographic characteristics, health history, and environmental exposures of study subjects. **Table S2.** Relationship between community composition variance using Adonis (PermANOVA) and a Canberra distance matrix of surveillance (healthy) samples (*n* = 47) and 58 measured variables detailed below. Note, only one individual was exposed to a cat at school and that same individual was the only subject with a positive corona virus (CoV) result in the Respiratory MultiCode Assay (RMA) viral screen, so the findings for these variables are inconclusive. Findings ranked by *P* value. **Table S3.** Genera enriched in children with a history of acute sinusitis (*n* = 14 shown in *gray* and those enriched in children without (*n* = 33) shown in white by Welch’s *t* test (*P* < 0.05, *q* < 0.05, ranked by P). **Table S4.** Taxa enriched in children with at least one episode of acute sinusitis during 1 year after sample collection (*n* = 7), shown in *gray* and those enriched in children who did not experience acute sinusitis (*n* = 33) during 1 year after sample collection by Welch’s *t* test, shown in white (*P* ≤ 0.05, *q* < 0.25, ranked by P). **Table S5.** Taxa detected on negative control PhyloChip and removed from further data analysis. (DOCX 76 kb) Additional file 2: Figure S1. Schematic of sampling and clinical follow-up time periods. Number in circle indicates the number of independent samples collected during that month, and the line indicates the time span of subsequent clinical monitoring. Colors indicate season. (TIFF 552 kb) Additional file 3: Figure S2. Normalized fluorescent intensity for Moraxella nonliquefaciens (eOTU 457) plotted for each subject showing significant differences (Welch’s *t* test, *P* ≤ 0.05) between (A) children with a prior history of acute sinusitis and those without a prior history of acute sinusitis as well as (B) children who developed acute sinusitis during the year following sample collection and those who did not. (TIFF 340 kb)
