# Supplementary material for: Nasopharyngeal microbiota composition of children is related to the frequency of upper respiratory infection and acute sinusitis
Source: Microbiome. 2016 Jul 1;4:34. doi: 10.1186/s40168-016-0179-9 (PMC4929776; doi:10.1186/s40168-016-0179-9)
Supplement: Additional file 1: Table S1. — Summary table of demographic characteristics, health history, and environmental exposures of study subjects. Table S2. Relationship between community composition variance using Adonis (PermANOVA) and a Canberra distance matrix of surveillance (healthy) samples (n = 47) and 58 measured variables detailed below. Note, only one individual was exposed to a cat at school and that same individual was the only subject with a positive corona virus (CoV) result in the Respiratory MultiCode Assay (RMA) viral screen, so the findings for these variables are inconclusive. Findings ranked by P value. Table S3. Genera enriched in children with a history of acute sinusitis (n = 14 shown in gray and those enriched in children without (n = 33) shown in white by Welch’s t test (P < 0.05, q < 0.05, ranked by P). Table S4. Taxa enriched in children with at least one episode of acute sinusitis during 1 year after sample collection (n = 7), shown in gray and those enriched in children who did not experience acute sinusitis (n = 33) during 1 year after sample collection by Welch’s t test, shown in white (P ≤ 0.05, q < 0.25, ranked by P). Table S5. Taxa detected on negative control PhyloChip and removed from further data analysis. (DOCX 76 kb) [file 40168_2016_179_MOESM1_ESM.docx]

**Supplemental Figure Legends.**

**Supplemental Figure S1.** Schematic of sampling and clinical follow up time periods. Number in circle indicates the number of independent samples collected during that month and the line indicates the time span of subsequent clinical monitoring. Colors indicate season.

**Supplemental Figure S2.**  Normalized fluorescent intensity for *Moraxella nonliquefaciens* (eOTU 457) plotted for each subject showing significant differences (Welch’s t-test, *P*≤0.05) between **(A)** children with a prior history of acute sinusitis and those without a prior history of acute sinusitis as well as **(B)** children who developed acute sinusitis during the year following sample collection and those who did not.

**Supplemental Tables**

**Table S1.** Summary table of demographic characteristics, health history, and environmental exposures of study subjects.

| **Characteristics** | **Number of Subjects** |
| --- | --- |
| **Maternal Education Level** |  |
| Grad/Prof | 15 |
| College Degree | 20 |
| Some College | 6 |
| Vo/Tech | 2 |
| High School or less | 2 |
| Grade School | 1 |
| Not reported | 1 |
| **Race** |  |
| American Indian or Alaska Native | 1 |
| Asian | 1 |
| African-American | 2 |
| Other | 2 |
| Caucasian | 41 |
| **Ethnicity** |  |
| Hispanic | 3 |
| Non-Hispanic | 43 |
| Not Reported | 1 |
| Age (years) mean, (range) | 5.64 (4-7) |
| Female Gender | 20 |
| Household size | 4.19 (2-6) |
| Live with other Children | 43 |
| Breastfed | 41 |
| Exposed to Tobacco | 2 |
| Exposed to Tobacco at Daycare | 1 |
| Animal Exposure (any) | 30 |
| Dog Exposure at Home | 17 |
| Dog Exposure at School | 3 |
| Cat Exposure at Home | 14 |
| Cat Exposure at School | 1 |
| Cow Exposure | 2 |
| Pig Exposure | 1 |
| Horse Exposure | 1 |
| Live on a Farm | 2 |
| Daycare | 40 |
| Wheezing Episodes | 6 |
| Family History of Asthma | 13 |
| Family History of Chronic Sinusitis | 13 |
| Family History of Allergy | 24 |
| **Acute Sinusitis (prior to enrollment in study)** | 14 |
| Mean Age of Initial Diagnosis (months) | 6.92 (5-19) |
| Mean Number of Episodes | 0.46 (0-3) |
| **Otitis Media (Prior to Study)** | 40 |
| Mean Age of Initial Diagnosis (months) | 5.875 (0-22) |
| Mean Number of Episodes | 3.4 (1-17) |
| **Medications** |  |
| Other | 5 |
| Antihistamines | 2 |
| Asthma Medications | 3 |
| Saline | 20 |
| Probiotics | 5 |
| **Allergy (any)** | 5 |
| Food Allergy | 2 |
| Drug Allergy | 3 |
| Eczema | 3 |
| Age of Diagnosis (months) | 3.8 (1,9) |
| Recent Vaccinations | 14 |
| **Viral Detection (by Respiratory Multicode Assay)** | |
| Corona Virus | 1 |
| Human Rhinovirus | 18 |
| Flu (A or B) | 2 |
| No Virus Detected | 26 |
| **Season of Sample Collection** |  |
| Spring (March 20-June 19) | 14 |
| Summer (June 20- September 19) | 10 |
| Fall (September 22-December 20) | 18 |
| Winter (December 21-March 19) | 5 |

| **Table S2.** Relationship between community composition variance using Adonis (PermANOVA) and a Canberra distance matrix of surveillance (healthy) samples (n=47) and 58 measured variables detailed below. Note, only one individual was exposed to a cat at school and that same individual was the only subject with a positive corona virus (CoV) result in the Respiratory MultiCode Assay (RMA) viral screen, so the findings for these variables are inconclusive. Findings ranked by P-value*.* | | |
| --- | --- | --- |
| **Variable** | ***P*** | ***R*^2^** |
| Season | 0.006 | 0.137 |
| Prior History of Acute Sinusitis | 0.009 | 0.070 |
| RMA Virus Detected: CoV | 0.022 | 0.048 |
| Cat Exposure at School | 0.022 | 0.048 |
| Tobacco Exposure at Home | 0.076 | 0.039 |
| Tobacco Exposure at Home or Daycare | 0.094 | 0.042 |
| Live on Farm | 0.113 | 0.035 |
| Days Between Sampling and First URI | 0.155 | 0.039 |
| Medications: Antihistamines | 0.165 | 0.044 |
| Family History of Allergy | 0.196 | 0.030 |
| Acute Sinusitis During One Year Following Sampling | 0.213 | 0.034 |
| Probiotics | 0.239 | 0.028 |
| Household Size (Grouped by number of members: 2-3, 4, 5-6) | 0.259 | 0.052 |
| Allergy, Other | 0.296 | 0.053 |
| Daycare | 0.299 | 0.025 |
| Otitis Media Prior History | 0.321 | 0.024 |
| Live With Other Children | 0.398 | 0.021 |
| Size of Household | 0.415 | 0.021 |
| Number of URI Over One Year | 0.425 | 0.024 |
| URI Within 30 Days of Sample Collection | 0.426 | 0.026 |
| Human Rhinovirus Detected (all types) | 0.479 | 0.019 |
| Medications: Saline | 0.490 | 0.019 |
| Number of Acute Sinusitis Episodes Prior to Sampling | 0.500 | 0.019 |
| Race | 0.501 | 0.084 |
| Animal Exposure (Any Type) | 0.507 | 0.019 |
| Tobacco Exposure at Daycare | 0.522 | 0.018 |
| Animal Exposure: Cat at Home | 0.523 | 0.018 |
| 16S Copies/uL (Bacterial Burden) | 0.529 | 0.050 |
| Animal Exposure: Pigs | 0.558 | 0.016 |
| Animal Exposure: Horses | 0.564 | 0.017 |
| RMA Virus Detected: Any | 0.570 | 0.017 |
| Family History of Asthma | 0.573 | 0.017 |
| Animal Exposure: Cows | 0.586 | 0.017 |
| Animal Exposure: Dog at Home | 0.594 | 0.036 |
| Allergy: Age of Diagnosis | 0.602 | 0.227 |
| Family History of Chronic Sinusitis | 0.618 | 0.016 |
| Daycare | 0.631 | 0.016 |
| Allergy Drug | 0.682 | 0.025 |
| Mother's Level of Education | 0.683 | 0.096 |
| URI Within 60 days of Sample Collection | 0.702 | 0.038 |
| Allergy: Any | 0.709 | 0.014 |
| Prior Acute Sinusitis: Age of Diagnosis | 0.711 | 0.049 |
| Allergy: Food | 0.716 | 0.025 |
| Allergy: Eczema | 0.717 | 0.023 |
| Animal Exposure: Dog at School | 0.728 | 0.014 |
| Prior Diagnosis of Otitis Media: Number of Times | 0.755 | 0.171 |
| Days Between Sample Collection and First Subsequent URI | 0.812 | 0.015 |
| Gender | 0.823 | 0.011 |
| Wheezing Episodes | 0.832 | 0.011 |
| Medications: Other | 0.845 | 0.011 |
| Age (Years) | 0.860 | 0.010 |
| Hispanic | 0.869 | 0.011 |
| Prior Diagnosis Otitis Media: Age of Diagnosis | 0.885 | 0.012 |
| Breastfed | 0.897 | 0.009 |
| Asthma | 0.910 | 0.010 |
| Recent Vaccinations | 0.941 | 0.008 |
| Medications: Asthma Medications | 0.956 | 0.014 |
| RMA Virus Detected: Flu (A or B) | 0.973 | 0.008 |

| **Table S3.** Genera enriched in children with a history of acute sinusitis (n=14 shown in grey and those enriched in children without (n=33) shown in white by Welch’s t-test (*P*<0.05, *q*<0.05, ranked by *P*) | | | | | |
| --- | --- | --- | --- | --- | --- |
| **eOTU** | **Phylum** | **Family** | **Genus** | ***P*** | ***q*** |
| 457 | *Proteobacteria* | *Moraxellaceae* | *Moraxella nonliquefaciens* | 0.020 | 0.035 |
| 276 | *Bacteroidetes* | *Prevotellaceae* | *Prevotella copri* | 0.0005 | 0.024 |
| 596 | *Bacteroidetes* | *RikenellaceaeII* | unclassified | 0.0008 | 0.024 |
| 406 | *Actinobacteria* | *Microbacteriaceae* | *Microbacterium* | 0.0011 | 0.024 |
| 1178 | *Firmicutes* | *Ruminococcaceae* | unclassified | 0.0012 | 0.024 |
| 913 | *Firmicutes* | *Clostridiaceae* | *Clostridium* | 0.0016 | 0.024 |
| 846 | *Bacteroidetes* | *Prevotellaceae* | *Prevotella* | 0.0016 | 0.024 |
| 257 | *Firmicutes* | *Ruminococcaceae* | *Subdoligranulum* | 0.0017 | 0.024 |
| 515 | *Firmicutes* | *Clostridiaceae* | *Clostridium* | 0.0017 | 0.024 |
| 1145 | *Tenericutes* | *Acholeplasmataceae* | *Candidatus Phytoplasma* | 0.0017 | 0.024 |
| 840 | *Firmicutes* | *Ruminococcaceae* | *Subdoligranulum* | 0.0017 | 0.024 |
| 1063 | *Bacteroidetes* | *Prevotellaceae* | *Prevotella* | 0.0017 | 0.024 |
| 268 | *Firmicutes* | *Clostridiaceae* | *Clostridium* | 0.0018 | 0.024 |
| 278 | *Proteobacteria* | *Sphingomonadaceae* | *Sphingomonas* | 0.0018 | 0.024 |
| 517 | *Bacteroidetes* | *Prevotellaceae* | *Prevotella* | 0.0020 | 0.024 |
| 804 | *Bacteroidetes* | *RikenellaceaeII* | unclassified | 0.0020 | 0.024 |
| 875 | *Firmicutes* | *Ruminococcaceae* | *Faecalibacterium prausnitzii* | 0.0023 | 0.024 |
| 1114 | *Proteobacteria* | *Enterobacteriaceae* | unclassified | 0.0023 | 0.024 |
| 873 | *Firmicutes* | *Lactobacillaceae* | *Lactobacillus* | 0.0023 | 0.024 |
| 813 | *Proteobacteria* | unclassified | unclassified | 0.0023 | 0.024 |
| 1184 | *Proteobacteria* | unclassified | unclassified | 0.0024 | 0.024 |
| 433 | *Firmicutes* | *Clostridiaceae* | *Clostridium* | 0.0024 | 0.024 |
| 1127 | *Firmicutes* | *Lachnospiraceae* | unclassified | 0.0025 | 0.024 |
| 836 | *Firmicutes* | *Lachnospiraceae* | unclassified | 0.0025 | 0.024 |
| 362 | *Firmicutes* | *Lachnospiraceae* | unclassified | 0.0026 | 0.024 |
| 426 | *Firmicutes* | unclassified | unclassified | 0.0026 | 0.024 |
| 1010 | *Firmicutes* | *Lachnospiraceae* | *Blautia* | 0.0026 | 0.024 |
| 793 | *Firmicutes* | *Lachnospiraceae* | unclassified | 0.0026 | 0.024 |
| 523 | *Bacteroidetes* | *Prevotellaceae* | *Prevotella* | 0.0027 | 0.024 |
| 1009 | *Firmicutes* | *Ruminococcaceae* | *Prevotella copri* | 0.0029 | 0.024 |
| 3 | *Firmicutes* | *Lachnospiraceae* | unclassified | 0.0030 | 0.024 |
| 1199 | *Proteobacteria* | *Vibrionaceae* | *Vibrio* | 0.0031 | 0.024 |
| 642 | *Bacteroidetes* | *Prevotellaceae* | *Prevotella* | 0.0031 | 0.024 |
| 435 | *Firmicutes* | *Lachnospiraceae* | unclassified | 0.0033 | 0.024 |
| 319 | *Proteobacteria* | *Comamonadaceae* | *Brachymonas denitrificans* | 0.0033 | 0.024 |
| 834 | *Firmicutes* | *Lachnospiraceae* | unclassified | 0.0033 | 0.024 |
| 1030 | *Firmicutes* | *Lachnospiraceae* | unclassified | 0.0035 | 0.024 |
| 1221 | *Proteobacteria* | *Rhodospirillaceae* | unclassified | 0.0036 | 0.024 |
| 1044 | *Firmicutes* | *Lachnospiraceae* | unclassified | 0.0036 | 0.024 |
| 805 | *Bacteroidetes* | *RikenellaceaeII* | unclassified | 0.0037 | 0.024 |
| 1183 | *Proteobacteria* | unclassified | unclassified | 0.0037 | 0.024 |
| 467 | *Proteobacteria* | *Coxiellaceae* | unclassified | 0.0038 | 0.024 |
| 124 | *Proteobacteria* | *Pseudomonadaceae* | *Pseudomonas* | 0.0039 | 0.024 |
| 337 | *Tenericutes* | *Erysipelotrichaceae* | *Bulleidia* p-1630-c5 | 0.0039 | 0.024 |
| 1051 | *Bacteroidetes* | *Bacteroidaceae* | *Bacteroides* | 0.0040 | 0.024 |
| 876 | *Firmicutes* | *Lachnospiraceae* | *Eubacterium* | 0.0041 | 0.024 |
| 506 | *Firmicutes* | *Lachnospiraceae* | unclassified | 0.0042 | 0.024 |
| 890 | *Tenericutes* | *Erysipelotrichaceae* | *PSB-M-3* | 0.0042 | 0.024 |
| 959 | *Firmicutes* | *Lachnospiraceae* | unclassified | 0.0043 | 0.024 |
| 520 | *Firmicutes* | *Ruminococcaceae* | *Streptococcus sinensis* | 0.0043 | 0.024 |
| 579 | *Firmicutes* | *Lachnospiraceae* | *Clostridium* | 0.0046 | 0.025 |
| 1277 | *Chloroflexi* | unclassified | unclassified | 0.0047 | 0.025 |
| 790 | *Firmicutes* | *Lachnospiraceae* | unclassified | 0.0051 | 0.025 |
| 874 | *Proteobacteria* | *Hyphomicrobiaceae* | *Rhodoplanes* | 0.0051 | 0.025 |
| 178 | *Firmicutes* | *Lachnospiraceae* | unclassified | 0.0051 | 0.025 |
| 928 | *Bacteroidetes* | *Prevotellaceae* | *Prevotella* | 0.0052 | 0.025 |
| 1039 | *Firmicutes* | *Ruminococcaceae* | unclassified | 0.0053 | 0.025 |
| 889 | *Planctomycetes* | *Isosphaeraceae* | unclassified | 0.0056 | 0.025 |
| 1028 | *Firmicutes* | *Lachnospiraceae* | unclassified | 0.0056 | 0.025 |
| 1040 | *Firmicutes* | *Lachnospiraceae* | unclassified | 0.0057 | 0.025 |
| 848 | *Bacteroidetes* | *Prevotellaceae* | *Prevotella* | 0.0059 | 0.025 |
| 508 | *Firmicutes* | *Lachnospiraceae* | unclassified | 0.0059 | 0.025 |
| 915 | *Tenericutes* | *Erysipelotrichaceae* | *Catenibacterium mitsuokai* | 0.0062 | 0.025 |
| 1228 | *Bacteroidetes* | unclassified | unclassified | 0.0062 | 0.025 |
| 835 | *Firmicutes* | *Lachnospiraceae* | unclassified | 0.0064 | 0.025 |
| 414 | *Firmicutes* | *Lachnospiraceae* | *Clostridium* | 0.0065 | 0.025 |
| 962 | *Firmicutes* | *Lachnospiraceae* | unclassified | 0.0067 | 0.025 |
| 171 | *Proteobacteria* | *Xanthomonadaceae* | *Xylella* | 0.0067 | 0.025 |
| 607 | *Actinobacteria* | *Nocardioidaceae* | *Actinopolymorpha* | 0.0069 | 0.025 |
| 925 | *Firmicutes* | *Lactobacillaceae* | *Lactobacillus* | 0.0070 | 0.025 |
| 1231 | *Bacteroidetes* | *Prevotellaceae* | *Prevotella* | 0.0070 | 0.025 |
| 454 | *Proteobacteria* | unclassified | *Sphingomonas azotifigens* | 0.0070 | 0.025 |
| 845 | *Bacteroidetes* | *Prevotellaceae* | *Prevotella* | 0.0071 | 0.025 |
| 237 | *Bacteroidetes* | *RikenellaceaeII* | unclassified | 0.0071 | 0.025 |
| 774 | *Firmicutes* | *Lachnospiraceae* | unclassified | 0.0071 | 0.025 |
| 1201 | *Proteobacteria* | *Rhodospirillaceae* | unclassified | 0.0071 | 0.025 |
| 578 | *Firmicutes* | *Ruminococcaceae* | *Faecalibacterium* | 0.0071 | 0.025 |
| 436 | *Firmicutes* | *Lachnospiraceae* | unclassified | 0.0071 | 0.025 |
| 1041 | *Firmicutes* | *Ruminococcaceae* | unclassified | 0.0072 | 0.025 |
| 105 | *Firmicutes* | *Streptococcaceae* | *Streptococcus* | 0.0072 | 0.025 |
| 1043 | *Firmicutes* | *Ruminococcaceae* | unclassified | 0.0074 | 0.025 |
| 1064 | *Bacteroidetes* | *Prevotellaceae* | *Prevotella* | 0.0074 | 0.025 |
| 150 | *Firmicutes* | *Bacillaceae* | *Brachymonas denitrificans* | 0.0075 | 0.025 |
| 932 | *Firmicutes* | *Ruminococcaceae* | *Faecalibacterium* | 0.0077 | 0.025 |
| 1083 | *Firmicutes* | unclassified | unclassified | 0.0078 | 0.025 |
| 324 | *Firmicutes* | *Lachnospiraceae* | unclassified | 0.0079 | 0.025 |
| 1121 | *Actinobacteria* | *Corynebacteriaceae* | *Corynebacterium* | 0.0080 | 0.025 |
| 8 | *Proteobacteria* | unclassified | unclassified | 0.0081 | 0.025 |
| 434 | *Firmicutes* | unclassified | unclassified | 0.0082 | 0.025 |
| 501 | *Chloroflexi* | *Anaerolinaceae* | unclassified | 0.0083 | 0.025 |
| 833 | *Firmicutes* | *Lachnospiraceae* | unclassified | 0.0084 | 0.025 |
| 470 | *Proteobacteria* | *Pseudomonadaceae* | *Pseudomonas* | 0.0085 | 0.025 |
| 157 | *Firmicutes* | unclassified | unclassified | 0.0085 | 0.025 |
| 1002 | *Actinobacteria* | unclassified | unclassified | 0.0086 | 0.025 |
| 1115 | *Actinobacteria* | unclassified | unclassified | 0.0092 | 0.027 |
| 829 | *Firmicutes* | *Lachnospiraceae* | unclassified | 0.0094 | 0.027 |
| 1015 | *Firmicutes* | *Lachnospiraceae* | unclassified | 0.0097 | 0.027 |
| 585 | *Firmicutes* | *Lachnospiraceae* | unclassified | 0.0097 | 0.027 |
| 152 | *Firmicutes* | *Lachnospiraceae* | unclassified | 0.0103 | 0.028 |
| 961 | *Firmicutes* | *Lachnospiraceae* | unclassified | 0.0103 | 0.028 |
| 988 | *Firmicutes* | *Lactobacillaceae* | *Lactobacillus* | 0.0103 | 0.028 |
| 593 | *Firmicutes* | *Lachnospiraceae* | unclassified | 0.0107 | 0.029 |
| 1246 | *Bacteroidetes* | *RikenellaceaeII* | unclassified | 0.0110 | 0.029 |
| 325 | *Firmicutes* | *Lachnospiraceae* | unclassified | 0.0112 | 0.029 |
| 791 | *Firmicutes* | *Lachnospiraceae* | unclassified | 0.0112 | 0.029 |
| 948 | *Firmicutes* | *Ruminococcaceae* | *Oscillospira* | 0.0114 | 0.029 |
| 425 | *Firmicutes* | *Staphylococcaceae* | *Staphylococcus* | 0.0117 | 0.030 |
| 1260 | *Tenericutes* | *Erysipelotrichaceae* | *Eubacterium biforme* | 0.0121 | 0.030 |
| 1090 | *Firmicutes* | *Lactobacillaceae* | *Lactobacillus* | 0.0121 | 0.030 |
| 154 | *Acidobacteria* | unclassified | unclassified | 0.0124 | 0.030 |
| 747 | *Proteobacteria* | *Desulfobacteraceae* | unclassified | 0.0124 | 0.030 |
| 1241 | *Bacteroidetes* | *RikenellaceaeII* | unclassified | 0.0125 | 0.030 |
| 801 | *Firmicutes* | *Lachnospiraceae* | unclassified | 0.0126 | 0.030 |
| 1177 | *Firmicutes* | *Lachnospiraceae* | unclassified | 0.0127 | 0.030 |
| 419 | *Proteobacteria* | *Comamonadaceae* | unclassified | 0.0128 | 0.030 |
| 912 | *Bacteroidetes* | *Sphingobacteriaceae* | *Parapedobacter* | 0.0130 | 0.030 |
| 1154 | *Firmicutes* | unclassified | unclassified | 0.0130 | 0.030 |
| 254 | *Firmicutes* | *Lachnospiraceae* | unclassified | 0.0132 | 0.030 |
| 1255 | *Tenericutes* | unclassified | unclassified | 0.0132 | 0.030 |
| 576 | *Firmicutes* | *Lactobacillaceae* | *Lactobacillus* | 0.0132 | 0.030 |
| 600 | *Firmicutes* | *Bacillaceae* | *Bacillus* | 0.0133 | 0.030 |
| 1150 | *Firmicutes* | *Lachnospiraceae* | unclassified | 0.0135 | 0.031 |
| 187 | *Firmicutes* | *Lachnospiraceae* | unclassified | 0.0136 | 0.031 |
| 323 | *Firmicutes* | *Lachnospiraceae* | unclassified | 0.0140 | 0.031 |
| 418 | *Cyanobacteria* | *Ulvophyceae* | unclassified | 0.0141 | 0.031 |
| 1035 | *Firmicutes* | unclassified | unclassified | 0.0142 | 0.031 |
| 1135 | *Proteobacteria* | unclassified | unclassified | 0.0148 | 0.032 |
| 176 | *Firmicutes* | *Lachnospiraceae* | unclassified | 0.0150 | 0.032 |
| 369 | *Firmicutes* | unclassified | unclassified | 0.0150 | 0.032 |
| 723 | *Proteobacteria* | unclassified | unclassified | 0.0151 | 0.032 |
| 580 | *Firmicutes* | *Lachnospiraceae* | *Clostridium* | 0.0157 | 0.033 |
| 844 | *Bacteroidetes* | *Prevotellaceae* | *Prevotella* | 0.0158 | 0.034 |
| 1186 | *Proteobacteria* | unclassified | unclassified | 0.0163 | 0.034 |
| 936 | *Proteobacteria* | *Sphingomonadaceae* | *Sphingomonas azotifigens* | 0.0166 | 0.034 |
| 368 | *Firmicutes* | *Veillonellaceae* | unclassified | 0.0166 | 0.034 |
| 159 | *Firmicutes* | *Bacillaceae* | unclassified | 0.0168 | 0.034 |
| 413 | *Firmicutes* | *Lachnospiraceae* | unclassified | 0.0169 | 0.034 |
| 312 | *Bacteroidetes* | *Prevotellaceae* | *Prevotella* | 0.0170 | 0.034 |
| 847 | *Bacteroidetes* | *Prevotellaceae* | *Prevotella* | 0.0171 | 0.034 |
| 1242 | *Bacteroidetes* | unclassified | unclassified | 0.0172 | 0.034 |
| 726 | *Proteobacteria* | unclassified | unclassified | 0.0172 | 0.034 |
| 810 | *Proteobacteria* | *Aeromonadaceae* | *Aeromonas* | 0.0174 | 0.034 |
| 225 | *Firmicutes* | *Lachnospiraceae* | unclassified | 0.0174 | 0.034 |
| 893 | *Planctomycetes* | *Planctomycetaceae* | *Planctomyces* | 0.0181 | 0.035 |
| 253 | *Firmicutes* | *Lachnospiraceae* | unclassified | 0.0183 | 0.035 |
| 293 | *Firmicutes* | *Streptococcaceae* | *Streptococcus* | 0.0183 | 0.035 |
| 1130 | *Firmicutes* | *Lachnospiraceae* | unclassified | 0.0184 | 0.035 |
| 175 | *Proteobacteria* | unclassified | unclassified | 0.0185 | 0.035 |
| 155 | *Firmicutes* | unclassified | unclassified | 0.0186 | 0.035 |
| 1165 | *Firmicutes* | *Lachnospiraceae* | unclassified | 0.0188 | 0.035 |
| 949 | *Firmicutes* | *Lachnospiraceae* | unclassified | 0.0191 | 0.035 |
| 386 | *Firmicutes* | *Bacillaceae* | *Bacillus* | 0.0194 | 0.035 |
| 1134 | *Firmicutes* | *Ruminococcaceae* | *Faecalibacterium* | 0.0195 | 0.035 |
| 892 | *Proteobacteria* | unclassified | unclassified | 0.0196 | 0.035 |
| 812 | *Proteobacteria* | unclassified | unclassified | 0.0199 | 0.035 |
| 1164 | *Firmicutes* | *Lactobacillaceae* | *Lactobacillus* | 0.0199 | 0.035 |
| 963 | *Firmicutes* | *Lachnospiraceae* | *Butyrivibrio* | 0.0203 | 0.035 |
| 947 | *Firmicutes* | *Ruminococcaceae* | unclassified | 0.0203 | 0.035 |
| 498 | *Verrucomicrobia* | *Verrucomicrobiaceae* | *Akkermansia* | 0.0203 | 0.035 |
| 366 | *Firmicutes* | *Ruminococcaceae* | unclassified | 0.0205 | 0.036 |
| 182 | *Firmicutes* | *Ruminococcaceae* | unclassified | 0.0206 | 0.036 |
| 73 | *Proteobacteria* | unclassified | unclassified | 0.0208 | 0.036 |
| 897 | *BRC1* | unclassified | unclassified | 0.0209 | 0.036 |
| 153 | *Firmicutes* | *Lachnospiraceae* | unclassified | 0.0211 | 0.036 |
| 986 | *Proteobacteria* | *Campylobacteraceae* | unclassified | 0.0212 | 0.036 |
| 616 | *Firmicutes* | unclassified | unclassified | 0.0213 | 0.036 |
| 229 | *Firmicutes* | *Lachnospiraceae* | unclassified | 0.0214 | 0.036 |
| 591 | *Firmicutes* | *Ruminococcaceae* | unclassified | 0.0215 | 0.036 |
| 1275 | *Chloroflexi* | *Anaerolinaceae* | unclassified | 0.0218 | 0.036 |
| 497 | *Acidobacteria* | *Acidobacteriaceae* | unclassified | 0.0219 | 0.036 |
| 181 | *Firmicutes* | *Ruminococcaceae* | unclassified | 0.0220 | 0.036 |
| 185 | *Firmicutes* | *Lachnospiraceae* | unclassified | 0.0221 | 0.036 |
| 179 | *Proteobacteria* | unclassified | unclassified | 0.0221 | 0.036 |
| 803 | *Bacteroidetes* | *RikenellaceaeII* | unclassified | 0.0222 | 0.036 |
| 887 | *Chloroflexi* | *Anaerolinaceae* | unclassified | 0.0223 | 0.036 |
| 1011 | *Firmicutes* | *Lachnospiraceae* | unclassified | 0.0224 | 0.036 |
| 540 | *Bacteroidetes* | *Prevotellaceae* | *Prevotella* | 0.0227 | 0.036 |
| 622 | *Firmicutes* | *Lachnospiraceae* | unclassified | 0.0228 | 0.036 |
| 255 | *Firmicutes* | *Lachnospiraceae* | unclassified | 0.0231 | 0.036 |
| 744 | *Firmicutes* | *Lachnospiraceae* | unclassified | 0.0231 | 0.036 |
| 100 | *Firmicutes* | unclassified | unclassified | 0.0232 | 0.036 |
| 326 | *Firmicutes* | *Lachnospiraceae* | *Clostridium* | 0.0232 | 0.036 |
| 424 | *Firmicutes* | unclassified | unclassified | 0.0236 | 0.036 |
| 992 | *Firmicutes* | *Streptococcaceae* | *Streptococcus* | 0.0241 | 0.037 |
| 378 | *Firmicutes* | *Staphylococcaceae* | *Staphylococcus* | 0.0243 | 0.037 |
| 572 | *Firmicutes* | *Ruminococcaceae* | *Faecalibacterium* | 0.0245 | 0.037 |
| 492 | *Tenericutes* | *Erysipelotrichaceae* | unclassified | 0.0248 | 0.037 |
| 174 | *Proteobacteria* | unclassified | unclassified | 0.0249 | 0.037 |
| 220 | *Firmicutes* | *Lachnospiraceae* | unclassified | 0.0250 | 0.037 |
| 1061 | *Bacteroidetes* | *RikenellaceaeII* | unclassified | 0.0251 | 0.037 |
| 7 | *Firmicutes* | *Lachnospiraceae* | unclassified | 0.0252 | 0.037 |
| 1218 | *Proteobacteria* | unclassified | unclassified | 0.0253 | 0.037 |
| 273 | *Firmicutes* | *Ruminococcaceae* | *Faecalibacterium* | 0.0254 | 0.037 |
| 275 | *Firmicutes* | *Veillonellaceae* | *Dialister* | 0.0255 | 0.037 |
| 727 | *Proteobacteria* | *Hyphomicrobiaceae* | *Rhodoplanes* | 0.0255 | 0.037 |
| 1234 | *Bacteroidetes* | unclassified | unclassified | 0.0256 | 0.037 |
| 807 | *Bacteroidetes* | *RikenellaceaeII* | unclassified | 0.0257 | 0.037 |
| 792 | *Firmicutes* | *Ruminococcaceae* | *Faecalibacterium* | 0.0257 | 0.037 |
| 373 | *Bacteroidetes* | unclassified | unclassified | 0.0260 | 0.037 |
| 1050 | *Bacteroidetes* | *RikenellaceaeII* | unclassified | 0.0260 | 0.037 |
| 480 | *Bacteroidetes* | *RikenellaceaeII* | unclassified | 0.0261 | 0.037 |
| 989 | *Firmicutes* | *Streptococcaceae* | *Streptococcus* | 0.0264 | 0.037 |
| 396 | *Proteobacteria* | unclassified | unclassified | 0.0266 | 0.037 |
| 1156 | *Firmicutes* | *Paenibacillaceae* | *Paenibacillus* | 0.0266 | 0.037 |
| 507 | *Firmicutes* | *Lachnospiraceae* | unclassified | 0.0267 | 0.037 |
| 1249 | *Cyanobacteria* | unclassified | unclassified | 0.0270 | 0.037 |
| 1233 | *Bacteroidetes* | *Flexibacteraceae* | unclassified | 0.0274 | 0.037 |
| 575 | *Firmicutes* | *Lachnospiraceae* | *Clostridium* sp. SS2/1 | 0.0278 | 0.038 |
| 66 | *Firmicutes* | *Lachnospiraceae* | unclassified | 0.0283 | 0.038 |
| 964 | *Firmicutes* | *Lactobacillaceae* | *Lactobacillus* | 0.0287 | 0.039 |
| 822 | *Firmicutes* | *Lachnospiraceae* | *Clostridium* | 0.0291 | 0.039 |
| 544 | *Firmicutes* | *Lachnospiraceae* | unclassified | 0.0293 | 0.039 |
| 1157 | *Firmicutes* | *Ruminococcaceae* | unclassified | 0.0295 | 0.039 |
| 776 | *Proteobacteria* | *Moraxellaceae* | *Acinetobacter* | 0.0297 | 0.039 |
| 1003 | *Actinobacteria* | *Corynebacteriaceae* | *Corynebacterium* | 0.0297 | 0.039 |
| 767 | *Firmicutes* | *Lactobacillaceae* | *Lactobacillus* | 0.0305 | 0.040 |
| 715 | *Firmicutes* | *Lachnospiraceae* | unclassified | 0.0310 | 0.041 |
| 644 | *Tenericutes* | unclassified | unclassified | 0.0313 | 0.041 |
| 1020 | *Firmicutes* | *Ruminococcaceae* | unclassified | 0.0315 | 0.041 |
| 570 | *Firmicutes* | *Lachnospiraceae* | unclassified | 0.0318 | 0.041 |
| 832 | *Firmicutes* | *Lachnospiraceae* | unclassified | 0.0319 | 0.041 |
| 568 | *Proteobacteria* | unclassified | unclassified | 0.0320 | 0.041 |
| 156 | *Proteobacteria* | *Halomonadaceae* | *Halomonas* | 0.0324 | 0.041 |
| 226 | *Firmicutes* | *Bacillaceae* | *Bacillus* | 0.0324 | 0.041 |
| 1019 | *Firmicutes* | *Lachnospiraceae* | unclassified | 0.0325 | 0.041 |
| 299 | *Firmicutes* | *Streptococcaceae* | *Streptococcus sinensis* | 0.0325 | 0.041 |
| 173 | *Firmicutes* | unclassified | unclassified | 0.0327 | 0.041 |
| 280 | *Firmicutes* | *Lactobacillaceae* | *Lactobacillus* | 0.0329 | 0.041 |
| 953 | *Firmicutes* | *Lachnospiraceae* | *Clostridium* | 0.0335 | 0.042 |
| 656 | *Firmicutes* | *Lachnospiraceae* | unclassified | 0.0339 | 0.042 |
| 327 | *Firmicutes* | *Lachnospiraceae* | unclassified | 0.0339 | 0.042 |
| 1222 | *Proteobacteria* | unclassified | unclassified | 0.0340 | 0.042 |
| 606 | *Proteobacteria* | *Enterobacteriaceae* | unclassified | 0.0341 | 0.042 |
| 541 | *Bacteroidetes* | *RikenellaceaeII* | unclassified | 0.0342 | 0.042 |
| 806 | *Bacteroidetes* | *RikenellaceaeII* | unclassified | 0.0346 | 0.042 |
| 735 | *Firmicutes* | unclassified | unclassified | 0.0348 | 0.042 |
| 1057 | *Bacteroidetes* | *Porphyromonadaceae* | *Porphyromonas* | 0.0353 | 0.043 |
| 711 | *Proteobacteria* | *Phyllobacteriaceae* | *Parvibaculum* | 0.0360 | 0.043 |
| 548 | *Firmicutes* | unclassified | unclassified | 0.0368 | 0.044 |
| 652 | *Proteobacteria* | *Halomonadaceae* | *Candidatus Portiera* | 0.0375 | 0.045 |
| 549 | *Firmicutes* | *Lachnospiraceae* | unclassified | 0.0378 | 0.045 |
| 184 | *Proteobacteria* | unclassified | unclassified | 0.0378 | 0.045 |
| 188 | *Proteobacteria* | *Shewanellaceae* | *Shewanella* | 0.0384 | 0.045 |
| 1084 | *Firmicutes* | *Staphylococcaceae* | *Staphylococcus* | 0.0387 | 0.045 |
| 707 | *Proteobacteria* | *Phyllobacteriaceae* | *Phyllobacterium* | 0.0388 | 0.045 |
| 1202 | *Proteobacteria* | unclassified | unclassified | 0.0389 | 0.045 |
| 911 | *Bacteroidetes* | *Flavobacteriaceae* | unclassified | 0.0392 | 0.045 |
| 717 | *Proteobacteria* | *Desulfobacteraceae* | unclassified | 0.0394 | 0.045 |
| 1272 | *Verrucomicrobia* | *Verrucomicrobiaceae* | unclassified | 0.0395 | 0.045 |
| 251 | *Firmicutes* | *Lachnospiraceae* | unclassified | 0.0395 | 0.045 |
| 1036 | *Firmicutes* | *Lachnospiraceae* | unclassified | 0.0399 | 0.045 |
| 601 | *Firmicutes* | unclassified | unclassified | 0.0402 | 0.045 |
| 973 | *Spirochaetes* | *Spirochaetaceae* | unclassified | 0.0406 | 0.045 |
| 158 | *Firmicutes* | unclassified | unclassified | 0.0407 | 0.045 |
| 954 | *Firmicutes* | *Lachnospiraceae* | unclassified | 0.0407 | 0.045 |
| 487 | *Cyanobacteria* | unclassified | unclassified | 0.0409 | 0.045 |
| 118 | *Firmicutes* | *Lachnospiraceae* | unclassified | 0.0416 | 0.046 |
| 329 | *Firmicutes* | *Lachnospiraceae* | unclassified | 0.0419 | 0.046 |
| 1276 | *Chloroflexi* | *Anaerolinaceae* | unclassified | 0.0422 | 0.046 |
| 224 | *Firmicutes* | *Ruminococcaceae* | unclassified | 0.0422 | 0.046 |
| 341 | *Firmicutes* | *Streptococcaceae* | *Streptococcus* | 0.0424 | 0.046 |
| 372 | *Bacteroidetes* | *RikenellaceaeII* | unclassified | 0.0426 | 0.046 |
| 566 | *Proteobacteria* | unclassified | unclassified | 0.0426 | 0.046 |
| 966 | *Firmicutes* | *Veillonellaceae* | unclassified | 0.0426 | 0.046 |
| 700 | *Firmicutes* | *Lachnospiraceae* | unclassified | 0.0428 | 0.046 |
| 9 | *Firmicutes* | *Lachnospiraceae* | unclassified | 0.0428 | 0.046 |
| 231 | *Firmicutes* | *Lachnospiraceae* | unclassified | 0.0429 | 0.046 |
| 1078 | *Firmicutes* | *Bacillaceae* | *Bacillus* | 0.0430 | 0.046 |
| 223 | *Bacteroidetes* | *RikenellaceaeII* | unclassified | 0.0431 | 0.046 |
| 1158 | *Firmicutes* | *Lachnospiraceae* | unclassified | 0.0436 | 0.046 |
| 714 | *Proteobacteria* | unclassified | unclassified | 0.0448 | 0.047 |
| 1278 | *Chloroflexi* | *Dehalogenimonaceae* | *Dehalogenimonas* | 0.0450 | 0.047 |
| 163 | *Firmicutes* | *Bacillaceae* | *Bacillus* | 0.0459 | 0.048 |
| 819 | *Firmicutes* | *Ruminococcaceae* | unclassified | 0.0466 | 0.049 |
| 742 | *Proteobacteria* | unclassified | unclassified | 0.0482 | 0.050 |
| 357 | *Actinobacteria* | unclassified | unclassified | 0.0484 | 0.050 |
| 61 | *Proteobacteria* | unclassified | unclassified | 0.0486 | 0.050 |
| 1219 | *Proteobacteria* | *Halomonadaceae* | *Halomonas* | 0.0495 | 0.051 |
| 197 | *Proteobacteria* | unclassified | unclassified | 0.0500 | 0.051 |

| **Table S4** Taxa enriched in children with at least one episode of acute sinusitis during one year after sample collection (n=7), shown in grey and those enriched in children who did not experience acute sinusitis (n=33) during one year after sample collection by Welch’s t-test, shown in white (*P*≤0.05, *q*<0.25, ranked by P) | | | | | | | | | | |  |
| --- | --- | --- | --- | --- | --- | --- | --- | --- | --- | --- | --- |
| **eOTU** | **Phylum** | | **Family** | | **Genus** | | **P** | | | **q** |  |
| 210 | | *Actinobacteria* | *Corynebacteriaceae* | *Corynebacterium* | | 0.05 | | | 0.24 | | |
| 457 | | *Proteobacteria* | *Moraxellaceae* | *Moraxella nonliquefaciens* | | | | 0.05 | 0.24 | | |
| 774 | *Firmicutes* | | *Lachnospiraceae* | | unclassified | | 0.0002 | | | 0.09 |  |
| 326 | *Firmicutes* | | *Lachnospiraceae* | | *Clostridium* | | 0.0003 | | | 0.09 |  |
| 1022 | *Firmicutes* | | unclassified | | unclassified | | 0.0005 | | | 0.09 |  |
| 426 | *Firmicutes* | | unclassified | | unclassified | | 0.0014 | | | 0.09 |  |
| 254 | *Firmicutes* | | *Lachnospiraceae* | | unclassified | | 0.0015 | | | 0.09 |  |
| 925 | *Firmicutes* | | *Lactobacillaceae* | | *Lactobacillus* | | 0.0018 | | | 0.09 |  |
| 332 | *Firmicutes* | | *Lachnospiraceae* | | *Clostridium* | | 0.0019 | | | 0.09 |  |
| 988 | *Firmicutes* | | *Lactobacillaceae* | | *Lactobacillus* | | 0.0020 | | | 0.09 |  |
| 1049 | *Bacteroidetes* | | unclassified | | unclassified | | 0.0020 | | | 0.09 |  |
| 138 | *Proteobacteria* | | unclassified | | unclassified | | 0.0022 | | | 0.09 |  |
| 255 | *Firmicutes* | | *Lachnospiraceae* | | unclassified | | 0.0023 | | | 0.09 |  |
| 275 | *Firmicutes* | | *Veillonellaceae* | | *Dialister* | | 0.0024 | | | 0.09 |  |
| 639 | *Bacteroidetes* | | *Prevotellaceae* | | *Prevotella* | | 0.0024 | | | 0.09 |  |
| 579 | *Firmicutes* | | *Lachnospiraceae* | | *Clostridium* | | 0.0027 | | | 0.09 |  |
| 324 | *Firmicutes* | | *Lachnospiraceae* | | unclassified | | 0.0028 | | | 0.09 |  |
| 760 | *Firmicutes* | | *Lachnospiraceae* | | unclassified | | 0.0028 | | | 0.09 |  |
| 585 | *Firmicutes* | | *Lachnospiraceae* | | unclassified | | 0.0029 | | | 0.09 |  |
| 954 | *Firmicutes* | | *Lachnospiraceae* | | unclassified | | 0.0030 | | | 0.09 |  |
| 819 | *Firmicutes* | | *Ruminococcaceae* | | unclassified | | 0.0031 | | | 0.09 |  |
| 752 | *Firmicutes* | | *Lachnospiraceae* | | unclassified | | 0.0031 | | | 0.09 |  |
| 958 | *Firmicutes* | | *Lachnospiraceae* | | *Clostridium* | | 0.0035 | | | 0.09 |  |
| 333 | *Firmicutes* | | *Lachnospiraceae* | | unclassified | | 0.0035 | | | 0.09 |  |
| 576 | *Firmicutes* | | *Lactobacillaceae* | | *Lactobacillus* | | 0.0036 | | | 0.09 |  |
| 271 | *Cyanobacteria* | | unclassified | | unclassified | | 0.0037 | | | 0.09 |  |
| 328 | *Firmicutes* | | *Lachnospiraceae* | | *Clostridium* | | 0.0038 | | | 0.09 |  |
| 325 | *Firmicutes* | | *Lachnospiraceae* | | unclassified | | 0.0039 | | | 0.09 |  |
| 751 | *Firmicutes* | | *Lachnospiraceae* | | unclassified | | 0.0040 | | | 0.09 |  |
| 580 | *Firmicutes* | | *Lachnospiraceae* | | *Clostridium* | | 0.0046 | | | 0.09 |  |
| 1023 | *Firmicutes* | | *Lachnospiraceae* | | unclassified | | 0.0046 | | | 0.09 |  |
| 1183 | *Proteobacteria* | | unclassified | | unclassified | | 0.0047 | | | 0.09 |  |
| 394 | *Proteobacteria* | | *Enterobacteriaceae* | | *Escherichia* | | 0.0048 | | | 0.09 |  |
| 578 | *Firmicutes* | | *Ruminococcaceae* | | *Faecalibacterium* | | 0.0049 | | | 0.09 |  |
| 448 | *Proteobacteria* | | unclassified | | unclassified | | 0.0049 | | | 0.09 |  |
| 845 | *Bacteroidetes* | | *Prevotellaceae* | | *Prevotella* | | 0.0061 | | | 0.11 |  |
| 339 | *Proteobacteria* | | *Vibrionaceae* | | *Photobacterium* | | 0.0062 | | | 0.11 |  |
| 267 | *Firmicutes* | | *Lachnospiraceae* | | *Coprococcus* | | 0.0063 | | | 0.11 |  |
| 137 | *Proteobacteria* | | *Rhodospirillaceae* | | unclassified | | 0.0067 | | | 0.11 |  |
| 912 | *Bacteroidetes* | | *Sphingobacteriaceae* | | *Parapedobacter* | | 0.0069 | | | 0.11 |  |
| 414 | *Firmicutes* | | *Lachnospiraceae* | | *Clostridium* | | 0.0072 | | | 0.12 |  |
| 833 | *Firmicutes* | | *Lachnospiraceae* | | unclassified | | 0.0079 | | | 0.12 |  |
| 1064 | *Bacteroidetes* | | *Prevotellaceae* | | *Prevotella* | | 0.0083 | | | 0.12 |  |
| 1193 | *Proteobacteria* | | unclassified | | unclassified | | 0.0083 | | | 0.12 |  |
| 1065 | *Firmicutes* | | *Bacillaceae* | | *Bacillus* | | 0.0088 | | | 0.12 |  |
| 801 | *Firmicutes* | | *Lachnospiraceae* | | unclassified | | 0.0090 | | | 0.12 |  |
| 366 | *Firmicutes* | | *Ruminococcaceae* | | unclassified | | 0.0091 | | | 0.12 |  |
| 142 | *Proteobacteria* | | unclassified | | unclassified | | 0.0092 | | | 0.12 |  |
| 1234 | *Bacteroidetes* | | unclassified | | unclassified | | 0.0095 | | | 0.12 |  |
| 828 | *Firmicutes* | | *Lachnospiraceae* | | unclassified | | 0.0097 | | | 0.12 |  |
| 756 | *Proteobacteria* | | *Alteromonadaceae* | | *Glaciecola* | | 0.0099 | | | 0.12 |  |
| 822 | *Firmicutes* | | *Lachnospiraceae* | | *Clostridium* | | 0.0099 | | | 0.12 |  |
| 411 | *Firmicutes* | | *Lachnospiraceae* | | unclassified | | 0.0103 | | | 0.12 |  |
| 1058 | *Bacteroidetes* | | *Prevotellaceae* | | *Prevotella* | | 0.0104 | | | 0.12 |  |
| 329 | *Firmicutes* | | *Lachnospiraceae* | | unclassified | | 0.0107 | | | 0.12 |  |
| 953 | *Firmicutes* | | *Lachnospiraceae* | | *Clostridium* | | 0.0107 | | | 0.12 |  |
| 791 | *Firmicutes* | | *Lachnospiraceae* | | unclassified | | 0.0110 | | | 0.12 |  |
| 1113 | *Proteobacteria* | | *Pasteurellaceae* | | *Haemophilus parainfluenzae* | | 0.0111 | | | 0.12 |  |
| 898 | *Firmicutes* | | *Veillonellaceae* | | *Veillonella ratti* | | 0.0112 | | | 0.12 |  |
| 932 | *Firmicutes* | | *Ruminococcaceae* | | *Faecalibacterium* | | 0.0113 | | | 0.12 |  |
| 948 | *Firmicutes* | | *Ruminococcaceae* | | *Oscillospira* | | 0.0113 | | | 0.12 |  |
| 314 | *Bacteroidetes* | | *Bacteroidaceae* | | *Bacteroides dorei* | | 0.0115 | | | 0.12 |  |
| 253 | *Firmicutes* | | *Lachnospiraceae* | | unclassified | | 0.0121 | | | 0.12 |  |
| 330 | *Firmicutes* | | *Lachnospiraceae* | | unclassified | | 0.0122 | | | 0.12 |  |
| 1028 | *Firmicutes* | | *Lachnospiraceae* | | unclassified | | 0.0127 | | | 0.12 |  |
| 425 | *Firmicutes* | | *Staphylococcaceae* | | *Staphylococcus* | | 0.0132 | | | 0.13 |  |
| 144 | *Proteobacteria* | | *Enterobacteriaceae* | | unclassified | | 0.0132 | | | 0.13 |  |
| 1233 | *Bacteroidetes* | | *Flexibacteraceae* | | unclassified | | 0.0140 | | | 0.13 |  |
| 962 | *Firmicutes* | | *Lachnospiraceae* | | unclassified | | 0.0142 | | | 0.13 |  |
| 450 | *Proteobacteria* | | *Enterobacteriaceae* | | unclassified | | 0.0147 | | | 0.13 |  |
| 517 | *Bacteroidetes* | | *Prevotellaceae* | | *Prevotella* | | 0.0152 | | | 0.14 |  |
| 834 | *Firmicutes* | | *Lachnospiraceae* | | unclassified | | 0.0153 | | | 0.14 |  |
| 1056 | *Bacteroidetes* | | *Prevotellaceae* | | *Prevotella* | | 0.0157 | | | 0.14 |  |
| 449 | *Proteobacteria* | | unclassified | | unclassified | | 0.0161 | | | 0.14 |  |
| 788 | *Bacteroidetes* | | unclassified | | unclassified | | 0.0161 | | | 0.14 |  |
| 453 | *Proteobacteria* | | unclassified | | unclassified | | 0.0165 | | | 0.14 |  |
| 282 | *Firmicutes* | | *Lachnospiraceae* | | *Roseburia* | | 0.0169 | | | 0.14 |  |
| 575 | *Firmicutes* | | *Lachnospiraceae* | | *Coprococcus* | | 0.0171 | | | 0.14 |  |
| 1110 | *Proteobacteria* | | unclassified | | unclassified | | 0.0175 | | | 0.14 |  |
| 1027 | *Firmicutes* | | *Lachnospiraceae* | | unclassified | | 0.0178 | | | 0.14 |  |
| 570 | *Firmicutes* | | *Lachnospiraceae* | | unclassified | | 0.0182 | | | 0.14 |  |
| 572 | *Firmicutes* | | *Ruminococcaceae* | | *Faecalibacterium* | | 0.0189 | | | 0.15 |  |
| 1061 | *Bacteroidetes* | | *RikenellaceaeII* | | unclassified | | 0.0194 | | | 0.15 |  |
| 337 | *Tenericutes* | | *Erysipelotrichaceae* | | *Bulleidia* p-1630-c5 | | 0.0198 | | | 0.15 |  |
| 124 | *Proteobacteria* | | *Pseudomonadaceae* | | *Pseudomonas* | | 0.0200 | | | 0.15 |  |
| 582 | *Firmicutes* | | *Lachnospiraceae* | | *Blautia* | | 0.0206 | | | 0.15 |  |
| 1044 | *Firmicutes* | | *Lachnospiraceae* | | unclassified | | 0.0206 | | | 0.15 |  |
| 601 | *Firmicutes* | | unclassified | | unclassified | | 0.0206 | | | 0.15 |  |
| 1060 | *Bacteroidetes* | | *RikenellaceaeII* | | unclassified | | 0.0208 | | | 0.15 |  |
| 34 | *Bacteroidetes* | | *Prevotellaceae* | | *Prevotella* | | 0.0209 | | | 0.15 |  |
| 32 | *Firmicutes* | | *Lachnospiraceae* | | *Blautia* | | 0.0212 | | | 0.15 |  |
| 955 | *Firmicutes* | | *Lachnospiraceae* | | *Clostridium* | | 0.0215 | | | 0.15 |  |
| 961 | *Firmicutes* | | *Lachnospiraceae* | | unclassified | | 0.0223 | | | 0.15 |  |
| 1232 | *Bacteroidetes* | | *RikenellaceaeII* | | unclassified | | 0.0227 | | | 0.15 |  |
| 256 | *Bacteroidetes* | | *RikenellaceaeII* | | unclassified | | 0.0236 | | | 0.16 |  |
| 946 | *Proteobacteria* | | *Enterobacteriaceae* | | unclassified | | 0.0242 | | | 0.16 |  |
| 1015 | *Firmicutes* | | *Lachnospiraceae* | | unclassified | | 0.0269 | | | 0.17 |  |
| 321 | *Proteobacteria* | | *Pasteurellaceae* | | unclassified | | 0.0270 | | | 0.17 |  |
| 1150 | *Firmicutes* | | *Lachnospiraceae* | | unclassified | | 0.0272 | | | 0.17 |  |
| 447 | *Proteobacteria* | | unclassified | | unclassified | | 0.0274 | | | 0.17 |  |
| 951 | *Firmicutes* | | *Lachnospiraceae* | | unclassified | | 0.0295 | | | 0.19 |  |
| 915 | *Tenericutes* | | *Erysipelotrichaceae* | | *Catenibacterium mitsuokai* | | 0.0300 | | | 0.19 |  |
| 829 | *Firmicutes* | | *Lachnospiraceae* | | unclassified | | 0.0301 | | | 0.19 |  |
| 1275 | *Chloroflexi* | | *Anaerolinaceae* | | unclassified | | 0.0311 | | | 0.19 |  |
| 960 | *Firmicutes* | | *Lachnospiraceae* | | unclassified | | 0.0314 | | | 0.19 |  |
| 528 | *Proteobacteria* | | unclassified | | unclassified | | 0.0321 | | | 0.19 |  |
| 1259 | *Tenericutes* | | unclassified | | unclassified | | 0.0322 | | | 0.19 |  |
| 956 | *Firmicutes* | | *Lachnospiraceae* | | *Coprococcus* | | 0.0322 | | | 0.19 |  |
| 675 | *Firmicutes* | | *Lachnospiraceae* | | unclassified | | 0.0323 | | | 0.19 |  |
| 571 | *Firmicutes* | | *Veillonellaceae* | | *Dialister invisus* | | 0.0323 | | | 0.19 |  |
| 790 | *Firmicutes* | | *Lachnospiraceae* | | unclassified | | 0.0338 | | | 0.19 |  |
| 100 | *Firmicutes* | | unclassified | | unclassified | | 0.0340 | | | 0.19 |  |
| 966 | *Firmicutes* | | *Veillonellaceae* | | unclassified | | 0.0341 | | | 0.19 |  |
| 280 | *Firmicutes* | | *Lactobacillaceae* | | *Lactobacillus* | | 0.0343 | | | 0.19 |  |
| 386 | *Firmicutes* | | *Bacillaceae* | | *Bacillus* | | 0.0356 | | | 0.20 |  |
| 744 | *Firmicutes* | | *Lachnospiraceae* | | unclassified | | 0.0363 | | | 0.20 |  |
| 862 | *Cyanobacteria* | | unclassified | | unclassified | | 0.0372 | | | 0.20 |  |
| 84 | *Firmicutes* | | *Veillonellaceae* | | *Veillonella ratti* | | 0.0385 | | | 0.21 |  |
| 616 | *Firmicutes* | | unclassified | | unclassified | | 0.0389 | | | 0.21 |  |
| 164 | *Firmicutes* | | *Lachnospiraceae* | | *Blautia* | | 0.0409 | | | 0.22 |  |
| 596 | *Bacteroidetes* | | *RikenellaceaeII* | | unclassified | | 0.0410 | | | 0.22 |  |
| 832 | *Firmicutes* | | *Lachnospiraceae* | | unclassified | | 0.0426 | | | 0.22 |  |
| 792 | *Firmicutes* | | *Ruminococcaceae* | | *Faecalibacterium* | | 0.0428 | | | 0.22 |  |
| 535 | *Proteobacteria* | | unclassified | | unclassified | | 0.0434 | | | 0.22 |  |
| 959 | *Firmicutes* | | *Lachnospiraceae* | | unclassified | | 0.0434 | | | 0.22 |  |
| 711 | *Proteobacteria* | | *Phyllobacteriaceae* | | *Parvibaculum* | | 0.0434 | | | 0.22 |  |
| 776 | *Proteobacteria* | | *Moraxellaceae* | | *Acinetobacter* | | 0.0437 | | | 0.22 |  |
| 876 | *Firmicutes* | | *Lachnospiraceae* | | *Eubacterium* | | 0.0452 | | | 0.23 |  |
| 393 | *Proteobacteria* | | *Pasteurellaceae* | | unclassified | | 0.0474 | | | 0.23 |  |
| 279 | *Firmicutes* | | *Lachnospiraceae* | | *Blautia* | | 0.0485 | | | 0.24 |  |
| 1063 | *Bacteroidetes* | | *Prevotellaceae* | | *Prevotella* | | 0.0491 | | | 0.24 |  |
| 433 | *Firmicutes* | | *Clostridiaceae* | | *Clostridium* | | 0.0501 | | | 0.24 |  |
| 1108 | *Proteobacteria* | | *Enterobacteriaceae* | | unclassified | | 0.0505 | | | 0.24 |  |
| 754 | *Proteobacteria* | | unclassified | | unclassified | | 0.0507 | | | 0.24 |  |
| 373 | *Bacteroidetes* | | unclassified | | unclassified | | 0.0513 | | | 0.24 |  |
| 155 | *Firmicutes* | | unclassified | | unclassified | | 0.0513 | | | 0.24 |  |
| 963 | *Firmicutes* | | *Lachnospiraceae* | | *Butyrivibrio* | | 0.0520 | | | 0.24 |  |
| 587 | *Firmicutes* | | *Lactobacillaceae* | | *Lactobacillus* | | 0.0521 | | | 0.24 |  |
| 986 | *Proteobacteria* | | *Campylobacteraceae* | | unclassified | | 0.0523 | | | 0.24 |  |
| 1164 | *Firmicutes* | | *Lactobacillaceae* | | *Lactobacillus* | | 0.0527 | | | 0.24 |  |
| 1189 | *Proteobacteria* | | *Aquabacteriaceae* | | *Aquabacterium* | | 0.0532 | | | 0.24 |  |
| 759 | *Proteobacteria* | | unclassified | | unclassified | | 0.0534 | | | 0.24 |  |
| 600 | *Firmicutes* | | *Bacillaceae* | | *Bacillus* | | 0.0541 | | | 0.24 |  |

**Table S5.** Taxa detected on negative control PhyloChip and removed from further data analysis.

| **eOTU** | **Phlyum** | **Famly** | **Genus** | **Species** |
| --- | --- | --- | --- | --- |
| 371 | *Bacteroidetes* | *Rikenellaceae II* | unclassified | unclassified |
| 1226 | *Bacteroidetes* | *Rikenellaceae II* | unclassified | unclassified |
| 134 | *Firmicutes* | *Lachnospiraceae* | unclassified | unclassified |
| 370 | *Firmicutes* | *Lachnospiraceae* | unclassified | unclassified |
| 648 | *Firmicutes* | *Lachnospiraceae* | unclassified | unclassified |
| 135 | *Proteobacteria* | *Moraxellaceae* | *Acinetobacter* | unclassified |
| 136 | *Proteobacteria* | *Pseudomonadaceae* | *Pseudomonas* | unclassified |
