# Supplementary figures and images for: Nasopharyngeal microbiota composition of children is related to the frequency of upper respiratory infection and acute sinusitis
Source: Microbiome. 2016 Jul 1;4:34. doi: 10.1186/s40168-016-0179-9 (PMC4929776; doi:10.1186/s40168-016-0179-9)

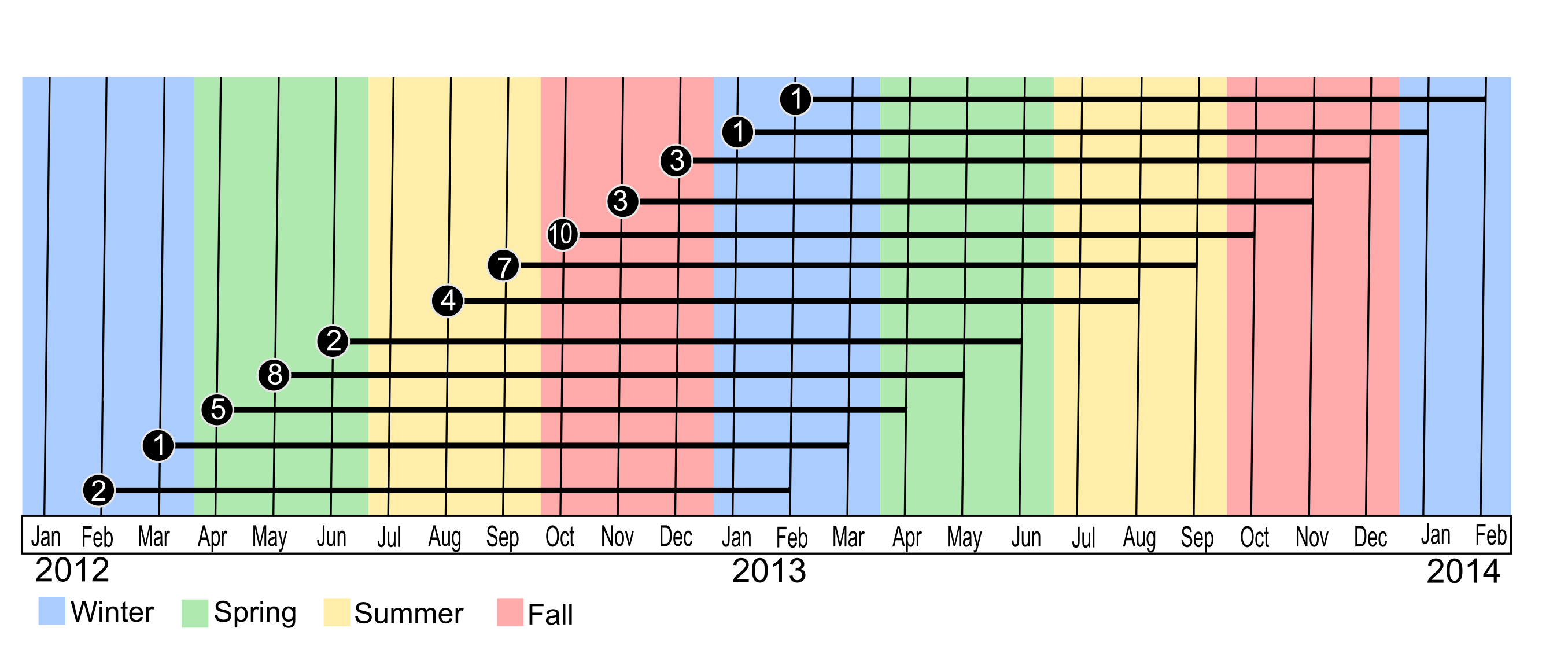

Supplement: Additional file 2: Figure S1. — Schematic of sampling and clinical follow-up time periods. Number in circle indicates the number of independent samples collected during that month, and the line indicates the time span of subsequent clinical monitoring. Colors indicate season. (TIFF 552 kb) [file 40168_2016_179_MOESM2_ESM.tiff]

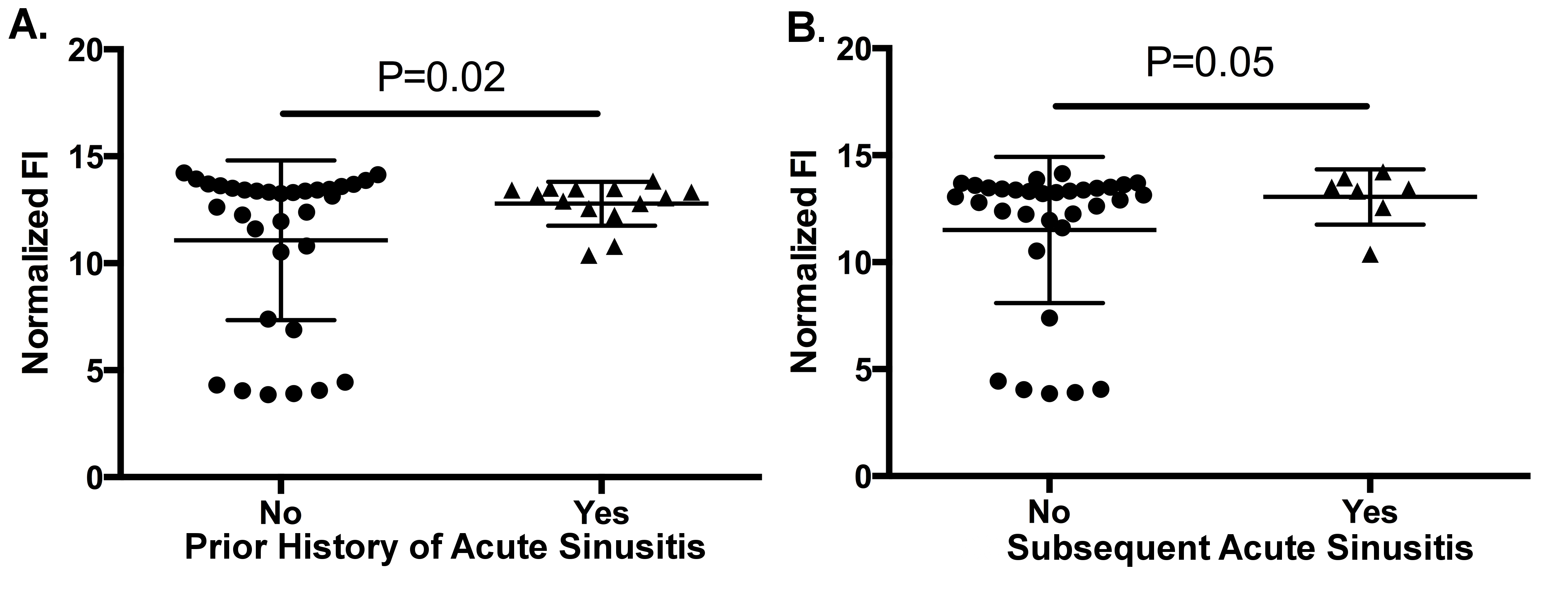

Supplement: Additional file 3: Figure S2. — Normalized fluorescent intensity for Moraxella nonliquefaciens (eOTU 457) plotted for each subject showing significant differences (Welch’s t test, P ≤ 0.05) between (A) children with a prior history of acute sinusitis and those without a prior history of acute sinusitis as well as (B) children who developed acute sinusitis during the year following sample collection and those who did not. (TIFF 340 kb) [file 40168_2016_179_MOESM3_ESM.tiff]
